# Active Rehabilitation Technologies for Post-Stroke Patients

**DOI:** 10.3390/bios16010020

**Published:** 2025-12-25

**Authors:** Hongbei Meng, Zihe Zhao, Shangru Li, Shengbo Wang, Jiacheng Wang, Canxi Yang, Chenyu Tang, Xuhang Chen, Xiaoxue Zhai, Yu Pan, Arokia Nathan, Peter Smielewski, Luigi G. Occhipinti, Shuo Gao

**Affiliations:** 1 School of Instrumentation and Optoelectronic Engineering, Beihang University, Beijing 100191, China; 22371194@buaa.edu.cn (H.M.); by1917059@buaa.edu.cn (Z.Z.); 21371329@buaa.edu.cn (S.L.); wangshengb@buaa.edu.cn (S.W.); 21371478@buaa.edu.cn (J.W.); 22371474@buaa.edu.cn (C.Y.); 2 Department of Engineering, University of Cambridge, Cambridge CB3 0FA, UK; ct631@cam.ac.uk (C.T.); lgo23@cam.ac.uk (L.G.O.); 3 Brain Physics Laboratory, Division of Neurosurgery, Department of Clinical Neurosciences, University of Cambridge, Cambridge CB2 0QQ, UK; xc369@cam.ac.uk; 4 Department of Physical Medicine and Rehabilitation, Beijing Tsinghua Changgung Hospital, Beijing 100084, China; zxxa02445@btch.edu.cn (X.Z.); panyu@btch.edu.cn (Y.P.); 5 Department of Engineering, Darwin College, University of Cambridge, Cambridge CB3 9EU, UK; an299@cam.ac.uk; 6 Wolfson Brain Imaging Centre, Addenbrooke’s Hospital, Cambridge CB3 0FA, UK; ps10011@cam.ac.uk

**Keywords:** stroke, active rehabilitation, neuroplasticity, intention recognition, feedback intervention, clinical practice

## Abstract

Neuroplasticity-based active movement opens an avenue for functional recovery in post-stroke patients. Active rehabilitation techniques have attracted wide attention based on their abilities to enhance patient involvement, facilitate precise personalized intervention, and provide comprehensive treatment via cross-domain approaches. Emerging evidence suggests that active rehabilitation methods can respond to patients’ motor intentions in real-time and significantly increase motivation and engagement, leading to efficient utilization of critical recovery windows and better rehabilitation outcomes. In this review, we focus on the physiological basis of active rehabilitation, including mechanisms of neuroplasticity, and discuss recent advances in intent detection and feedback devices. We also examine treatment options for different stages of stroke recovery, providing a comprehensive reference for engineers to design optimized rehabilitation techniques and for clinicians to select appropriate rehabilitation protocols. These developments create new opportunities to improve the lives of stroke patients and offer greater hope for their recovery.

## 1. Introduction

Stroke is a leading cause of death and adult-acquired disability worldwide, characterized by sudden neurological deficits due to vascular obstruction, rupture, or hemorrhage. Common mechanisms include blood clots that interrupt cerebral blood flow, leading to ischemia and abrupt neuronal death from oxygen deprivation [1]. Despite advancements in acute medical care reducing stroke-related mortality, a growing number of individuals live with permanent impairments post-stroke. Estimates suggest that nearly 1% of the global population experiences stroke sequelae [2], including impaired motor control [3], cognitive deficits [4,5], speech difficulties [6], and altered emotional states [7]. Chronic motor dysfunction affects up to 30% of stroke survivors [2], making hemiplegia one of the most prevalent disabling conditions post-stroke. Consequently, restoration of motor function is a primary focus of rehabilitation efforts.

Traditional stroke rehabilitation methods, such as physical therapy, occupational therapy [8], and traditional Chinese medicine, involve patients passively receiving interventions. These methods are often limited by insufficient dosage, low patient engagement, and a lack of objective feedback, which impede the advancement of motor function in stroke survivors and preclude the optimal exploitation of the critical window of recovery. Over the past few decades, substantial progress has been made in developing active rehabilitation techniques that leverage the principles of neuroplasticity—the brain’s ability to reorganize and form new neural connections. In contrast to the unidirectional feedback output of passive rehabilitation, active rehabilitation emphasizes a closed-loop “central-peripheral -central” model of rehabilitation. This model improves patient engagement and motivation, as well as the individualization and standardization of feedback interventions. These novel rehabilitation techniques employ external devices that are controlled by the patient’s subjective motor intentions, thereby markedly enhancing the immediacy of feedback interventions. To report the state-of-the-art works to researchers in this field, numerical reviews have been generated. For example, Everard et al. [9] summarize the impact of new technologies, including virtual reality (VR), robot-assisted therapy (RAT), and telerehabilitation (TR) on upper extremity (UE) motor function and daily activities in stroke patients, comparing and assessing the influence of rehabilitation design, motor impairment severity, and treatment duration. Bui et al. [10] provide a comprehensive evaluation of the potential of VR in promoting UE rehabilitation after stroke and explore the added value of VR as a therapeutic tool, especially when used in combination with traditional rehabilitation techniques. Liu et al. [11] focus on patient needs and review the actuation and control strategies of hand function rehabilitation robots and VR virtual task positioning to improve the effectiveness of active rehabilitation training. Yang et al. [12] analyze the feasibility of using electroencephalogram (EEG) and electromyography (EMG) signals for state-of-the-art motor function rehabilitation and demonstrate that EEG and EMG activities provide complementary information for detecting movement intention, i.e., the accuracy of hybrid brain-machine interface (hBMI) is significantly higher than that of a single biological signal. Hribernik et al. [13] review the applications of real-time biomechanical feedback (BMF) systems in sports and rehabilitation, emphasizing the different building blocks of BMF systems and their technological basis. As summarized in Table 1, most existing reviews focus on specific technology classes (such as VR, robotics, EEG/EMG-based BCIs or telerehabilitation) and typically adopt either a primarily engineering or a primarily clinical perspective. By contrast, relatively few overviews integrate neuroplasticity mechanisms, intention-detection and feedback technologies, and phase-specific rehabilitation needs within a single framework. This gap motivates the more mechanistic and cross-stage synthesis developed in the present review.

Emerging evidence suggests that active rehabilitation techniques can effectively promote neuroplastic changes, leading to improved functional outcomes for stroke survivors. These methods facilitate repetitive, task-specific training, active patient participation, and precise measurement of functional improvement. However, most existing reviews focus on the technical efficacy of individual methodologies, often from a singular medical or engineering perspective. This may entail either focusing exclusively on patient needs and efficacy while neglecting the system’s drive and control strategies, or developing an efficient control system but failing to consider the patient’s recovery period. This fragmented approach may overlook the crucial alignment between technological innovations and clinical applications, posing challenges in integrating engineering solutions with patient-specific rehabilitation stages and subjective experiences. In many cases, it is challenging for medical professionals to articulate their needs in engineering terms, and vice versa. To this end, this review article builds a bridge between doctors and engineers by explaining the physiological basis of active rehabilitation, intention detection, and feedback technology, combined with the patient’s subjective feelings and stroke recovery period to develop the required rehabilitation system.

**Figure 1:**
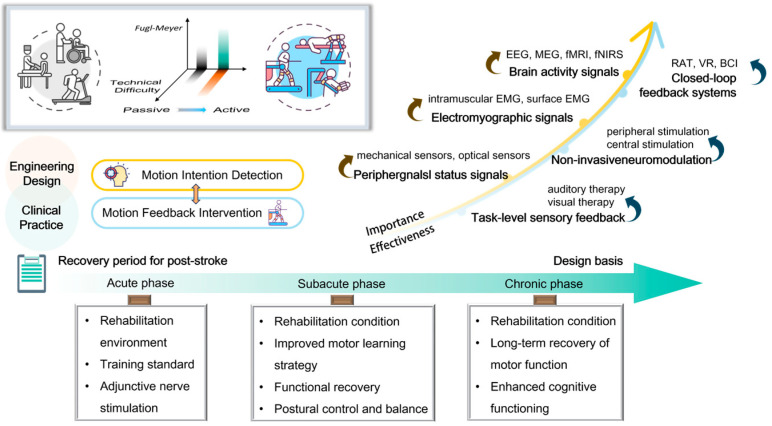
Conceptual roadmap for active rehabilitation after stroke. The diagram emphasizes the shift in stroke rehabilitation from passive exercise to intention-driven, feedback-rich practices. It highlights the synergy between engineering design and clinical practice, and introduces a series of movement intention detection and feedback strategies, then guides the key design priorities for selecting appropriate active rehabilitation programs at each stage.

In this review, we discuss the physiological basis of active rehabilitation, focusing on mechanisms of neuroplasticity that underlie motor recovery after stroke. We examine recent advances in intention detection and feedback technologies—the two principal components of active rehabilitation systems. Finally, we will present a synthesis of the various active rehabilitation programs applicable to the different recovery phases of stroke. This provides a framework for healthcare professionals to design appropriate approaches based on the patient’s objective rehabilitation conditions and specific circumstances as shown in Figure 1. Our objective is the realization of the most efficacious design or combination of treatment programs, intending to advance the use and development of active rehabilitation techniques in clinical practice. To this end, the review does not merely collate available techniques, but organizes them along a mechanistic–technological–clinical axis. Starting from activity-dependent forms of neuroplasticity that have been demonstrated in experimental and human studies, we discuss how different classes of motion-intention detection and feedback interventions are designed to interact with these processes, and how far this has been substantiated by clinical evidence at the hyperacute, acute, subacute and chronic stages of stroke rehabilitation.

## 2. Physiological Basis of Active Rehabilitation

Neuroplasticity represents a fundamental physiological foundation for the restoration of voluntary motor abilities in individuals who have experienced a stroke. Among the various rehabilitation strategies, active rehabilitation facilitates neuroplasticity at multiple levels of the neuroaxis and expedites functional reorganization of the brain. It is therefore important to recognize the subjective intentions of the patient and to provide them with accurate sensory feedback. Our findings indicate that the body’s biological signals are capable of visualizing motor intentions. By applying specific sensory feedback stimuli, the patient’s neural circuits are established and repaired, and neuroplasticity is promoted in both directions. In alignment with the understanding of the mechanism of brain neuroplasticity, it is essential to effectively adjust and enhance the implementation of active rehabilitation to facilitate the recovery of patients’ damaged functions.

### 2.1. Mechanisms of Neuroplasticity

**Figure 2:**
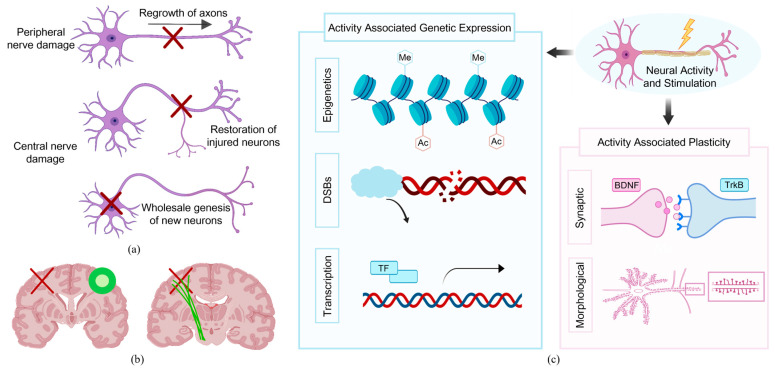
Examples of neuroplasticity. (**a**) Mechanisms of neuroplasticity in healthy individuals. (**b**) Neuroplasticity after stroke. (**c**) Neural activity can have a broad impact on the presentation of neurons by altering gene expression and their structure and function.

Neuroplasticity refers to the nervous system’s ability to modify and regenerate in response to new information or injury [28]. At this stage, neural networks undergo reorganization and functional restoration [29,30,31,32], including the restoration of the contralateral somatosensory system [33,34], improvement in the structural integrity of the affected corticospinal tracts [35], and restoration of the interhemispheric sensorimotor cortical network [36], as shown in Figure 2a,b. Such spontaneous plastic changes may also be associated with pathways including structural remodeling at the synapse, axon, and dendrite levels [37], nerve regeneration [38], release of neurotrophic factors [39], and the activation, migration, and differentiation of endogenous neural stem cells [40].

Over the past several decades, researchers have made seminal contributions to our understanding of synaptic function through the application of electrophysiological techniques to the analysis of synaptic morphology, dendritic and dendritic spine growth, and axonal distributions. This body of work has illuminated the regulatory roles of nucleolar proteins, such as CaMKIV and CREB, in gene transcription processes associated with learning and memory in long-term depression (LTD) [41], as well as the impact of the endogenous sensitive phosphatase calcineurin in long-term potentiation (LTP) on the strengthening of new synaptic connections [42].

Recent studies have revealed novel mechanisms of transcriptional and translational regulation associated with neuronal activity [43,44,45]. These mechanisms involve immediate early genes (IEGs) [46], epigenetic modifications, and other factors. One such mechanism includes the induction of double-strand DNA breaks (DSBs) at specific genomic locations, which enhances transcriptional stability and amplification. This process rapidly initiates transcriptional changes through various mechanisms, including transcription factors and IEGs, which ultimately alter the overall gene expression profile of neurons. Neuronal activity and external stimuli trigger these transcriptional changes, which in turn modulate responses to neurotransmitters and other signaling pathways, leading to morphological changes and synaptic plasticity, as shown in Figure 2c. A focal point of these studies is the brain-derived neurotrophic factor (BDNF) and its receptors, which play a crucial role in synaptic morphogenesis and are involved in neuronal differentiation, survival, migration, and dendritic spine development. Accordingly, factors that regulate BDNF expression, such as DNA methylation, histone modifications, and the transcription of related microRNAs, have also been included as key areas of research.

**Figure 3:**
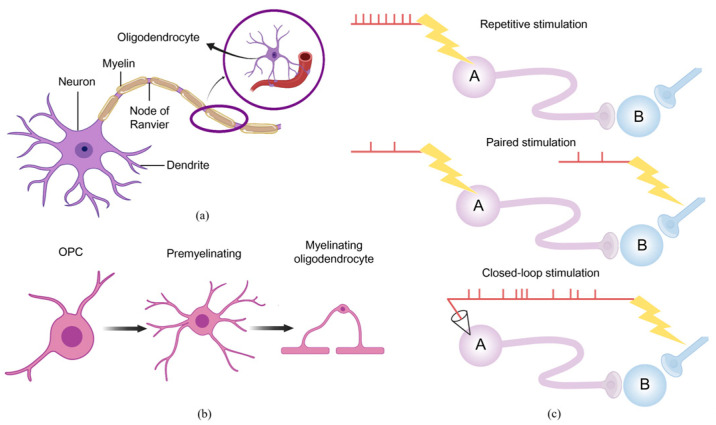
Myelin plasticity and Hebb Theory. (**a**) Myelin and the node of Ranvier. (**b**) Development of oligodendrocytes. (**c**) Protocols for inducing plasticity according to Hebb’s rule.

Furthermore, the conduction time of neural circuits represents a significant factor in the context of neuroplasticity. Myelin can facilitate the optimization of the temporal organization of neural pathways [47,48], thereby enhancing the efficiency of information transmission. For example, oligodendrocytes transmit high-speed pulses through jumping conduction after myelin formation on axons in Figure 3a. Additionally, oligodendrocyte progenitors (OPCs) differentiate into multiple early myelin oligodendrocytes and subsequently mature into myelin oligodendrocytes. OPCs constitute the majority of mitotic cells in the adult brain. Mature oligodendrocytes are capable of forming myelin fragments on multiple axons simultaneously. Their two-way communication with the peripheral neural network can facilitate an increase in myelin proteins, thereby improving nerve regeneration and further promoting synaptic plasticity [49], as shown in Figure 3b. Consequently, brain neuroplasticity continuously repairs and strengthens itself spatially and temporally, enabling the remodeling of cortical maps and the compensation for local functional impairments.

### 2.2. Contribution of Active Rehabilitation to Neuroplasticity

Active rehabilitation has been demonstrated to facilitate plasticity at multiple levels of the neuraxis, and scientists have commenced investigations into its contribution to neuroplasticity in animals. In rodent and feline models of thoracic spinal cord injury (SCI), researchers have observed at the cellular level that active exercise reduces the expression of inhibitory molecules, increases the expression of neurotrophic factors, and alters the electrophysiological properties of the lumbar spine enlargement. These changes have the potential to attenuate some of the spontaneous maladaptive plasticity and facilitate adaptive structural and functional plasticity [50]. In a small preclinical study in a rat stroke model [51], researchers record single and multi-neuron activity during active exercise and observe that active rehabilitation significantly promoted the reorganization of the cortical somatotopic map. This reorganization leads to heightened activity of the associated neurons, and further elevation of BDNF levels at the level of skeletal muscle and spinal cord innervation. Concurrently, in accordance with Hebb’s theory that repetitive stimulation of postsynaptic neurons by presynaptic neurons is essential for enhancing synaptic efficacy [52], Figure 3c illustrates one method of inducing plasticity, whereby repeated stimulation of neuron A results in associated presynaptic and postsynaptic activity due to the strong activation of a single pathway. The activation of neurons in A, occurring in response to a single stimulus, can be matched to stimulate neurons in B, resulting in robust output. By utilizing a closed-loop configuration, the recorded activity from the neurons in A serves as an endogenous trigger to initiate stimulation in B. Consequently, the reinforcing influence of sustained exercise on functional reorganization observed in the experimental setting is further augmented, prompting the integration of intensive repetitive training into the treatment protocol for SCI patients [53].

In addition to the effects observed in animal experiments, during active rehabilitation such as robotic exercise in patients, scientists have found that motor neurons in the human cortex can “learn” to control additional muscles and generate new movements as a result of stimulation [54], which suggests that new neural circuits can be established. Similarly, the expression of BDNF and its related receptor tyrosine kinase B (TrkB) continues to be upregulated. As previously described, this molecular mechanism may be a major regulator of the biochemical cascade of neuroplasticity [50]. Given the specificity of BDNF, the effects of different active rehabilitation programs may vary. Therefore, active rehabilitation may facilitate the repair of spinal circuits in the cortex, descending supraspinal motor pathways, and the caudal part of the injury. It may also result in the reorganization of joint motor function, the promotion of muscle function and the activation of multiple afferent stimulation modalities, and an enhancement of neuroplasticity efficacy via upregulation of BDNF.

### 2.3. Intent Detection Signaling Mechanisms

Depending on the source of the acquired biosignal, there are two main modalities for human motion intent recognition: biomechanical-based signals and bioelectrical-based signals. Biomechanical-based signals, such as gyroscopes, accelerometers, and capacitance information, can be used for intention estimation via inverse kinematics with traditional physical sensors. However, the mechanical information is generated after the human body initiates motion, resulting in a discernible time lag in information extraction. In contrast, bioelectrical signals, such as electrooculogram (EOG) signals, EEG signals, and EMG signals, which are generated before a patient’s intention to move has manifested, are an important means of detecting that intention. Therefore, we analyze the physiological mechanisms that underpin these common bioelectrical signals.

**Figure 4:**
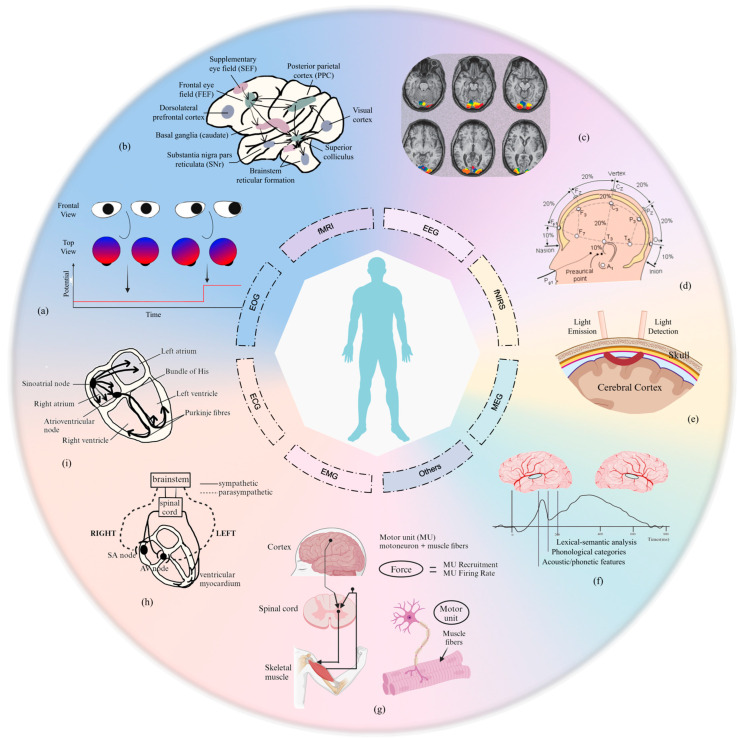
Intention detection signal mechanism. (**a**) Illustrations of eye movements and changes in electrical potential around the eyes. (**b**) Main neural structures involved in saccade control. (**c**) Functional magnetic resonance imaging (fMRI) sections of occipital visual areas show activity when stimulated with visual patterns. Functional magnetic resonance imaging provided courtesy of Jeffrey Anderson, University of Utah School of Med-icine. (**d**) The 10–20 International system seen from the left and above the head. (**e**) During near-infrared (NIR) spectrum analysis, photons are in-troduced at the scalp and pass through the tissue where they are either scattered or absorbed. Photodetectors can measure the photons that follow a banana-shaped path back to the skin’s surface. This method provides a relatively predictable quantity of photons for measurement. (**f**) Speech perception activity is detected bilaterally in the superior temporal cortex at the brain level. The illustration shows the temporal process of speech perception in the upper temporal cortex based on neurophysiological recording. (**g**) A motor unit (MU) consists of a spinal motoneuron and its innervated muscle fibers. (**h**) Pathways of sympathetic and parasympathetic innervation of the heart. (**i**) Progression of the electrical signal through a healthy heart.

The eyes serve as the primary conduit for information exchange and interaction. Firstly, the tracking of eye movement utilizing the position of the gaze point or the movement of the eyeball concerning the head, including aspects such as gaze, eye hopping, smooth following movements, blinking, and nystagmus, can be employed to hypothesize about the cognitive activity of the patient. Figure 4b illustrates the principal neural structures that are implicated in the control of saccadic eye movements. Secondly, eye movements also contribute to the potential difference between the cornea and the retina. The potential increases when the cornea approaches the electrode and decreases when the cornea moves in the opposite direction, as shown in Figure 4a [55]. Therefore, the recognition of human intentions is also facilitated by the detection of EOG in various eye movement patterns.

Electroencephalogram signals are classified into two categories: spontaneous EEG signals and evoked EEG signals. This classification is based on the underlying generation mechanism. Spontaneous EEG signals are typically classified into the delta (δ), theta (θ), alpha (α), beta (β), gamma (γ), and mu-rhythm categories based on their spontaneous frequency. The generating conditions and signal characteristics of different frequency bands reflect varying intentions. Concurrently, the amplitude of the mu-rhythm and Beta wave in the contralateral motor-sensory cortex region of the brain is markedly diminished in response to limb movement on one side of the body or imagined limb movement, while the corresponding rhythm on the ipsilateral side is markedly enhanced, resulting in Event-Related Desynchronization (ERD) and Event-Related Synchronization (ERS) phenomena [56]. The occurrence of ERD/ERS phenomena in the cerebral cortex simultaneously and the considerable amplitude difference constitute a significant basis for identification. Evoked EEG signals are electrical signals generated in specific regions of the cerebral cortex in response to external stimuli received by the subject. These include, for example, P300 and visual evoked potentials (VEP). P300 is an event-related potential that reaches its peak amplitude of 300 ms after the subject is stimulated by a visual or acoustic stimulus [57]. VEP elicits specific electrical signals in the occipital lobe of the brain, particularly in areas 17, 18, and 19, in response to visual stimuli such as light or graphic images (Figure 4c). These stimuli elicit changes in potential in the aforementioned areas of the brain [58].

Electromyographic signals are used to visualize states such as muscle activity or relaxation, as shown in Figure 4g. Action potentials are generated when muscle fibers contract and the amplitude of the EMG signal is proportional to the number of muscle fibers involved in muscle activity at the same time. When the action potentials generated by multiple muscle fibers are superimposed and mixed in the muscle, the real-time state of the muscle can be captured and evaluated by EMG.

Some indirect signals, such as functional near-infrared spectroscopy (fNIRS), can reflect changes in brain blood oxygen content (Figure 4e), which are also coupled with neural activity. When the human body generates subjective intentions or receives external stimuli, the brain transitions from a resting state to an excited state. During this transition, functional regions of the brain are activated, resulting in hemodynamic responses [59]. In these activated brain regions, the metabolic balance of blood and oxygen is disrupted, neuronal activity increases, and cerebral oxygen consumption rises.

Moreover, signals such as electrocardiogram (ECG), fMRI, magnetoencephalography (MEG), galvanic skin response (GSR), and skin temperature measurement (SKT) can be employed to indirectly discern the patient’s intention to move.

### 2.4. Facilitation of Neuroplasticity by Rehabilitation Intervention

The concept of enhancing plasticity in the injured central nervous system to optimize motor recovery is referred to as a “top-down” approach [60], such as plasticizing drugs or Non-Invasive Brain Stimulation (NIBS) techniques [61]. These methods are typically classified as either invasive or non-invasive, depending on the necessity for surgical implantation. The former typically exhibits superior target specificity but necessitates invasive surgical procedures, whereas the latter circumvents the risks of infection associated with invasive surgery, exhibits mild side effects, is well tolerated by patients, but displays poor targeting, and often necessitates multiple repetitions of the stimulation. Currently, electromagnetic stimulation techniques, including TMS, TES, deep brain electrical stimulation (DBS), and neuromuscular electrical stimulation (NMES), are the most prevalent.

**Figure 5:**
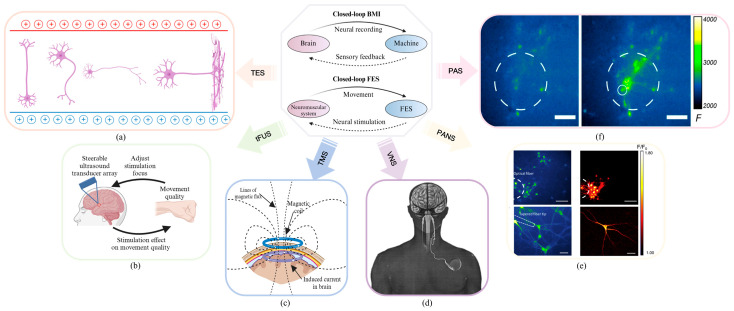
Mechanism of rehabilitation intervention. (**a**) The effect of neuronal compartment orientation on transcranial electrical stimulation (TES) induced excitability. Four idealized neurons (the soma, dendrites, axon initial segment, and the axon tree) are shown with different orientations relative to the induced electric field. (**b**) Illustration of the application of transcranial focused ultrasound stimulation (tFUS) to determine the brain regions involved in specific behaviors and behavioral disorders. (**c**) Illustration of the current direction in magnetic coil and induced current in the brain during transcranial magnetic stimulation (TMS). (**d**) An illustration of how the vagus nerve is stimulated by the NeuroCybernetic Prosthesis System. Reprinted with permission from Ref. [64]. Copyright 2000, Elsevier. (**e**) Spatial distribution of the activation of the neurons induced by PANs. (**f**) During photoacoustic stimulation, carbon nanotubes (CNT) functionalized with polyethylene glycol (PEG) are embedded into silk fibroin to create biocompatible and soft photoacoustic materials, known as CNT/silk scaffolds. These scaffolds have been shown to stimulate neuronal activity through photoacoustic waves. The illustration shows representative fluorescence images of the neuron cultures on the CNT/silk films before and after PA stimulation, with the dashed circles showing the illumination area.

Meanwhile, novel stimulation approaches, such as light and ultrasound stimulation, are still in the research and development phase. These novel rehabilitation interventions share the common features of targeted modulation of central nervous system activity and induction of neuroplasticity, which may contribute to their therapeutic benefits [62,63]. Figure 5 illustrates the typical mechanisms of rehabilitation intervention.

Transcranial magnetic stimulation utilizes a time-varying magnetic field to stimulate the cerebral cortex, resulting in the generation of induced currents that alter the action potentials of cortical nerve cells (Figure 5a). This consequently affects the metabolism and neuroelectric activity within the brain. The modulation of aberrant cortical network patterns or the normalization of cortical excitability via repetitive transcranial magnetic stimulation (rTMS) can facilitate neurological recovery [65]. The implementation of specific rTMS protocols can result in long-duration potentiation and inhibitory changes [66,67], which can effectively assist in rehabilitation by selecting appropriate stimulation targets and interstimulation intervals.

Transcranial electrical stimulation can induce neuromodulation [68], with different types of electrical discharges exerting varying degrees of influence on cortical networks. Electrical stimulation parameters determine the categorization of TES, which includes transcranial direct current stimulation (tDCS), transcranial alternating current stimulation (tACS), transcranial random noise current stimulation (tRNS), and transcranial pulsed electrical stimulation (tPCS). Among these, tACS generates periodic electric field changes in the brain through the application of different frequency stimulation patterns, affecting the synchronization and desynchronization of the electrical activity of brain nerve cells, thereby regulating brain function. This has the potential to facilitate functional rehabilitation following a stroke [69].

Invasive electrical stimulation has been demonstrated to enhance the excitability of undamaged neural circuits, thereby promoting the control or activation of neural networks. Additionally, it has been shown to affect the connectivity and repair of developmental tracts, as well as to maintain and improve spinal circuits that are not preserved after injury [70]. For example, DBS entails the implantation of stimulating electrodes at particular targets in the brain, which are stimulated at high frequencies to achieve deep neurostimulation: the stimulation of structures such as the internal capsule affects pathways originating from the primary motor area and extending to the spinal motor neurons, thereby promoting motor rehabilitation. Similarly, stimulation of deep cerebellar structures also affects motor function via the cerebellopontine-cerebellar pathway [71].

The application of transcutaneous electrical nerve stimulation (TENS) to peripheral nerves has been demonstrated to elicit functional activation of motor-related circuits along the nerve conduction pathway to the corresponding cortical regions, thereby activating cerebral function and evoking spinal control of muscle activity. Studies have shown that both epidural and transcutaneous electrical stimulation activates primary afferent fibers within multiple posterior roots [71]. The most probable mechanism of stimulation is to enhance the excitability of spinal networks through tonic activation of dorsal root afferent fibers. This, in turn, brings interneurons and motoneurons closer to motor thresholds and therefore more prone to respond to the restricted post-injury descending drive [71,72,73].

Vagus nerve stimulation (VNS) refers to the stimulation of the left cervical vagus nerve using a commercial device, the NCP System (Figure 5d) [64]. VNS has been demonstrated to alleviate the insufficient blood supply in ischemic stroke, inhibit the release of inflammatory cytokines such as tumor necrosis factor and interleukin, and produce anti-inflammatory effects. This reduces the inflammatory response around the stroke lesion and allows for the modulation of the sensory-motor rehabilitation of ischemic stroke in the acute and subacute phases.

Transcranial focused ultrasound stimulation has the potential to temporarily disrupt the blood–brain barrier, increasing its permeability. This allows for the passage of BDNF through the blood–brain barrier, thereby improving neurological symptoms.

The use of red to NIR light in the therapy known as Photobiomodulation (PBM) represents an emerging innovative approach targeting various neurological and psychological disorders [74]. Red/NIR light has been demonstrated to stimulate the mitochondrial respiratory chain complex IV, thereby increasing adenosine triphosphate (ATP) synthesis. The absorption of light by ion channels has been shown to result in the release of Ca2+, which in turn activates transcription factors and initiates gene expression. PBM therapy has been observed to enhance neuronal metabolism, stimulate anti-inflammatory, anti-apoptotic, and antioxidant responses, and promote neurogenesis and synaptic regeneration. This technology has the potential to be a valuable addition to the treatment of conditions such as stroke, traumatic brain injury, and depression.

In addition, epidural stimulation, photoacoustic nanotransducers (PANs), and novel photoacoustic stimulation (Figure 5f) have been demonstrated to be beneficial in replacing, enhancing, and remodeling motor and sensory functions, thereby improving the efficacy of neural plasticity.

### 2.5. Needs and Challenges

Although neuroplasticity is a crucial factor in the recovery of motor functions, it is important to note that not all forms of neuroplasticity can facilitate this process. For example, a bilateral interhemispheric inhibition (IHI) imbalance is one of the most prevalent negative adaptive plasticity modifications after a stroke [75]. It is crucial to determine whether to prioritize the enhancement of cortical excitability on the affected side or the promotion of compensatory excitation and the expansion of neural networks in the peripheral and contralateral hemispheres, depending on the specific patient, the location of the injury, and the extent of damage. Furthermore, the issue of active rehabilitation remains to be addressed due to the restricted control of neural circuits following a central nervous system (CNS) injury, which consequently limits the number and variety of neurons activated with a subjective intention. In other words, activity-dependent mechanisms that could, in principle, support recovery are constrained by the reduced pool of recruitable neurons and by the risk of reinforcing maladaptive patterns such as pathological synergies or disuse.

In addition, feedback stimulation of patients is also contingent upon individual variability, abiotic variations within the microenvironment, and stimulation deficits, to determine the optimal dosage and site for neurons to acquire accurate information at the cellular and neural pathway levels [76]. Recently, researchers have generated new strategies to selectively activate neural circuits using transgenic manipulation of neurons, which requires further empirical investigation. Meanwhile, the recognition of motor intention based on bioelectrical signals is also susceptible to several factors, including electrode movement, changes in skin impedance, artefactual interference, and localized specific discharges in the injured brain. Consequently, there is a need to further explore the development of accurate recognition algorithms that can maintain reliable performance over time and across different clinical environments.

Therefore, post-stroke neuroplasticity is both an opportunity and a constraint for engineering design. On the one hand, activity-dependent mechanisms provide a physiological basis for functional improvement if the appropriate neural circuits are repeatedly and specifically engaged. On the other hand, the effectiveness of such pairing depends on several critical factors: precise temporal alignment between intention-related signals and delivered feedback, adequate yet safe training intensity, careful choice of stimulation targets in the lesioned and contralesional networks, and substantial inter-individual variability driven by lesion characteristics, spontaneous reorganization and fluctuations in arousal, attention, fatigue and motivation. These mechanistic and practical constraints mean that determining “optimal” stimulation parameters and target sites cannot rely on a one-size-fits-all protocol, but requires accurate intention-detection algorithms, adaptive control strategies and careful clinical monitoring.

From an engineering perspective, the neuroplasticity processes can be grouped into three families of mechanisms that are directly relevant for system design: (i) activity-dependent synaptic plasticity and BDNF-related signaling; (ii) neuromodulator-gated, transcriptionally regulated plasticity, including activity-induced DNA breaks and epigenetic modifications; and (iii) myelin and structural plasticity that tune conduction time and the temporal precision of neural interactions. In the following sections, we use this classification to discuss how different families of rehabilitation technologies aim to create training conditions that engage one or more of these mechanism classes.

## 3. Techniques and Devices for Active Rehabilitation

**Figure 6:**
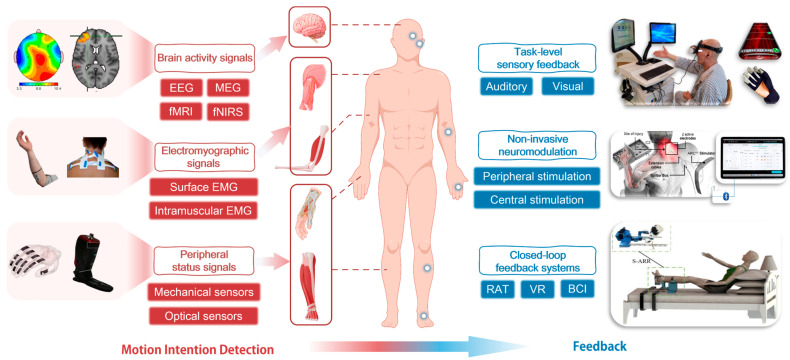
Motion-intention detection and feedback modalities in active rehabilitation systems. The diagram groups motion-intention detection signals according to their physiological origin and illustrates typical measurement methods. These signals are linked to the corresponding neural and musculoskeletal structures and then to three families of feedback interventions considered in this review: task-level sensory feedback, non-invasive neuromodulation and closed-loop feedback systems.

The recovery of motor function following a stroke is associated with neuroplasticity, which encompasses the formation of new neuronal connections, the acquisition of new abilities, and compensation for damage. Active rehabilitation, which involves combining the intention to perform movement with sensorimotor feedback, is more likely to induce neuroplasticity in the motor cortex, resulting in superior rehabilitation outcomes compared to passive exercise or limb stimulation alone. It is therefore evident that accurate recognition of patients’ motor intentions and sensory feedback based on them represent the key technologies to achieve active rehabilitation based on neuroplasticity. This chapter will therefore review the detection techniques used to identify patients’ motor intentions in active rehabilitation and the feedback provided to assist patients in realizing motion perception. The schematic diagram of this section is shown in Figure 6.

### 3.1. Motion Intention Detection Technology and Device

Given the pivotal role of patients’ subjective consciousness in active rehabilitation [77], it is of paramount importance to develop accurate methods for detecting patients’ intentions and enabling machines to comprehend them [78]. We group commonly used intention-detection signals according to their physiological origin and their timing with respect to movement onset. First, peripheral status signals derived from kinematic, force, pressure and optical measurements around the limb and assistive device primarily reflect the state of the limb and device during movement execution. Second, electromyographic signals capture muscle activation at the neuromuscular level and therefore correspond to the initiation of movement. Third, brain activity signals such as EEG, MEG and hemodynamic methods can index motor planning and imagery before any overt movement occurs. This organization aims to guide the engineering implementation of intention detection.

#### 3.1.1. Peripheral Status Signals for Movement Execution

Peripheral status signals are obtained from inertial, force, pressure and optical sensors attached to, or observing, the limb and the assistive device. They primarily represent the mechanical and kinematic state of the limb–device system during movement execution, and therefore reflect the consequences of motor commands rather than the underlying motor intention. However, these sensors measure the movement information that has already occurred, which results in a delay when compared with electromyographic signals and brain signals [79]. Due to the limitations of the insufficient correlation between peripheral status signals and intentions, they are generally limited to movement pattern recognition. Direct utilization of these sensors for MI tasks may result in lag and distortion.

**Figure 7:**
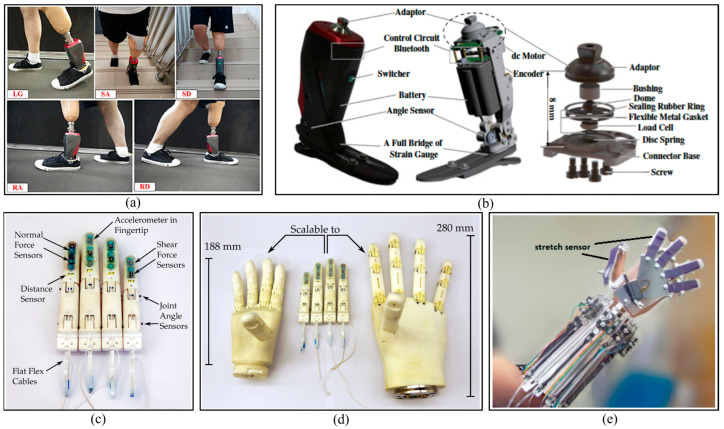
(**a**) Subject wears the robotic prosthesis to finish five locomotion modes: level ground walking (LG), stair ascending (SA), stair descending (SD), ramp ascending (RA), and ramp descending (RD). Reprinted with permission from Ref. [85]. Copyright 2020, Frontiers. (**b**) Robotic transtibial prosthesis prototype. Reprinted with permission from Ref. [86]. Copyright 2019, Cambridge University Press. (**c**) The four manufactured demonstrators derived from the scalable model. (**d**) A comparison of the physical demonstrators with the most recent Karlsruhe Institute of Technology (KIT) Prosthetic Hand (left) and the robotic KIT ARMAR-6 Hand (right). Reprinted with permission from Ref. [90]. Copyright 2019, MDPI. (**e**) Power-assistive hand exoskeleton. Reprinted with permission from Ref. [91]. Copyright 2018, MDPI.

Mechanical sensors may be employed to measure biomechanical signals. Such devices include goniometers, accelerometers, gyroscopes, magnetometers, inertial measurement units (IMUs), and strain gauges. As these sensors are closely related to movement, they are frequently employed in conjunction with one another [80,81,82,83]. Concerning the detection of lower extremity (LE) intentions, Zhang et al. [84] employ the use of accelerometers to identify movement patterns and put forth a novel methodology to circumvent the periodic detection and inter-cycle misalignment issues. Xu and Wang [85] advance a real-time onboard training model strategy for robot prosthesis recognition based on IMU (IMU is straightforward to integrate with prostheses, as shown in Figure 7a), with only two IMUs achieving a recognition accuracy of over 93%. Feng and Wang [86] also propose a gait recognition method based on strain gauges in conjunction with sensors such as accelerometers and angular velocity sensors (Figure 7b). In addition to LE rehabilitation, UE hand exoskeleton devices are also commonly employed in rehabilitation, as shown in Figure 7, in which mechanical sensors also serve the function of determining position and force to prevent active devices from causing injury to patients [87,88,89].

Optical sensors have a wide range of applications in the field of medicine, and can also be used to measure a variety of indicators. In the realm of movement prediction, a set of common indicators may prove pertinent, including heart rate (HR), respiratory rate (RR), and blood pressure, among others. Although traditional mechanical sensors are capable of measuring relevant indicators, for instance, the conventional approach to blood pressure measurement employs an inflatable pressure cuff, this technique is challenging to integrate and miniaturize following the requisite specifications. In contrast, optical sensors possess exemplary metrological characteristics and are not susceptible to electromagnetic interference, thereby ensuring electrical safety and excellent miniaturization. These characteristics render optical sensors an excellent advanced solution for the utilization of wearable devices and the general medical monitoring of physiological parameters. Currently, approximately 15% of the wearable device market is based on optical sensors, a figure that is still growing [92,93].

From a signal-processing perspective, peripheral status signals are typically processed through a pattern-recognition pipeline that combines feature extraction on joint kinematics, temporal gait parameters and interaction forces with statistical or machine-learning models. For locomotion-mode and gait-phase recognition in exoskeletons and prostheses, rule-based finite state machines are often used to encode plausible transitions between walking states, while hidden Markov models and Kalman filters support continuous state estimation and trajectory smoothing. On top of these temporal models, supervised classifiers such as support vector machines, random forests and gradient-boosted decision trees are widely used to classify multi-sensor feature vectors derived from inertial measurement units and plantar pressure sensors [94,95]. More recent work has explored convolutional, recurrent and hybrid CNN–LSTM architectures that operate directly on raw or minimally processed IMU and pressure time series to capture long-range spatiotemporal dependencies in gait patterns and locomotion transitions [96].

#### 3.1.2. Electromyographic Signals for Muscle Activation

**Figure 8:**
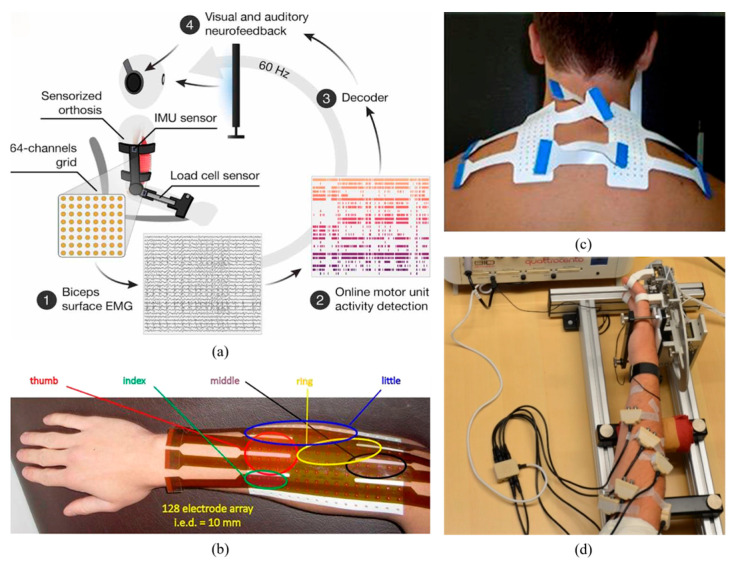
(**a**) Schematic of the neuromuscular–machine interface (NMI) built on surface EMG (sEMG) used to enable individual MU control of the biceps brachii. Reprinted with permission from Ref. [103]. Copyright 2021, IOP Publishing Ltd. (**b**) Monitoring the activity zones of the finger extensor muscles. Reprinted with permission from Ref. [104]. Copyright 2020, Frontiers. (**c**) Example of two electrode grids applied to the trapezius muscle. Reprinted with permission from Ref. [104]. Copyright 2020, Frontiers. (**d**) Simultaneous recording of intramuscular EMG (iEMG) of hand muscle contraction and generated force. Reprinted with permission from Ref. [105]. Copyright 2022, MDPI.

The muscles are the actuators of the sensory-motor system and contain a wealth of movement information [97]. Consequently, EMG signals can be used to directly and profoundly reflect human movement intentions [98,99,100,101,102]. Depending on the detection location, EMG signals can be divided into iEMG and sEMG. Figure 8 illustrates the detection of disparate muscle activities via sensors.

Intramuscular EMG signals are detected through implanting needles or wires, and offer the advantages of high selectivity (enabling the analysis of signals from single motor units or even single muscle fibers) and deep muscle detection (such as the genioglossus muscle) [106]. Consequently, they are extensively employed in neuromuscular evaluation for clinical intervention. Nevertheless, the technology is markedly invasive and carries the potential for patient harm and compromised local detection efficacy. Furthermore, the deployment of iEMG is confined to certified professionals and is conducted under rigorous supervision, both of which constrain its overall accessibility [107,108].

Surface EMG sensors are capable of recording the electrical potentials generated by muscle cells. The electrodes of sEMG sensors facilitate a chemical equilibrium between the detection surface and the human skin through electrolytic conduction, thereby enabling the flow of current into the electrodes [109]. Therefore, in actual measurements, the electrodes must be firmly attached to the skin of the limb. In comparison to iMEG, surface electromyographic signals provide a non-invasive detection method that is more acceptable to patients and researchers. However, there are some issues and limitations associated with sEMG sensing methods. The measured sEMG signals are weak and unstable [110], and they are susceptible to contamination by motion artifacts, muscle fatigue, electrode displacement, and crosstalk between nearby muscles.

A novel non-contact capacitive sensing method has recently been proposed for the measurement of muscle relaxation and contraction [111]. The human limb and a metal electrode can be regarded as two electrodes of a capacitor. A dielectric layer (e.g., a silicone layer) is placed between the metal electrode and the human limb, and the metal electrode comprises an equivalent capacitor. During limb movement, contraction and relaxation cause changes in the relative area and distance between the two electrodes. By measuring the charging and discharging cycle time, capacitance signals are recorded, and their feasibility has been demonstrated [112].

Most EMG-based intention-detection systems follow a well-established pattern-recognition pipeline. Raw EMG signals are first band-pass filtered and segmented into short analysis windows, from which time- and frequency-domain features—such as mean absolute value, root mean square, waveform length, zero-crossing counts, autoregressive coefficients and power in specific frequency bands—are extracted. These feature vectors are then fed into classifiers such as linear discriminant analysis, support vector machines, k-nearest neighbors, random forests or multilayer perceptrons to discriminate between discrete hand gestures or locomotion modes, or into regression models for continuous torque and joint-angle estimation [113]. High-density surface EMG and multi-channel sEMG arrays further enable spatially informed decoding, where convolutional and hybrid CNN–LSTM architectures operate on 2D or 3D “EMG images” to capture both spatial and temporal activation patterns. Recent studies have reported high recognition accuracies for complex gesture sets and have begun to address practical issues such as electrode shift and signal unreliability using attention-based and robust deep learning models [114,115,116].

#### 3.1.3. Brain Activity Signals for Motor Intention

The detection of cerebral signals is frequently regarded as being inextricably linked to the patient’s motor intentions and motor imagery. In post-stroke rehabilitation, research therefore concentrates on identifying and analyzing such motor-related brain activity using sophisticated imaging and electrophysiological modalities. Among these techniques, structural imaging can reveal the anatomical structure and morphology of the brain; however, it is not closely related to intention detection. Functional imaging and electrophysiological methods are therefore the focus of this section, and the main approaches include EEG, MEG, fMRI and fNIRS. These techniques offer distinct advantages in terms of spatial and temporal resolution: EEG and MEG provide millisecond-level temporal information, whereas fMRI and fNIRS offer higher spatial specificity. In the context of stroke, they are used to obtain spatiotemporal information regarding abnormal neural activity in motor-related networks and to monitor how these networks reorganize during recovery.

In a similar manner to muscle signals, invasive techniques yield superior outcomes but also entail elevated risks. The most fundamental approach is electrocorticography (ECoG), which offers the benefit of high-quality spatial and temporal resolution. However, due to the inherent surgical risks and the gradual deterioration of recorded data, invasive electrodes present significant disadvantages [117]. In contrast, non-invasive techniques have become more prevalent in human subjects.

**Figure 9:**
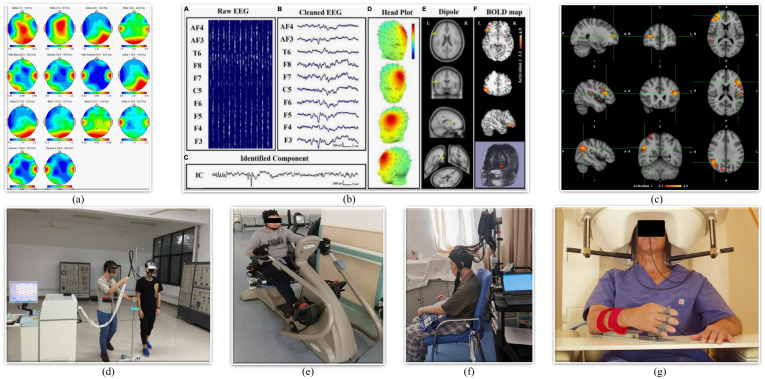
(**a**) An EEG power spectrum of a 23-year-old healthy volunteer [31]. (**b**) Simultaneous EEG-fMRI analysis on a patient with focal epilepsy [31]. (**c**) Illustration of three epileptic foci (each row) based on fMRI data analysis in three patients with different refractory focal epilepsy. Reprinted with permission from Ref. [31]. Copyright 2022, Frontiers. (**d**) A researcher walking with the subject carrying the weight of the fNIRS cables. Reprinted with permission from Ref. [124]. Copyright 2021, Technology Press. (**e**) Limb linkage rehabilitation training study using fNIRS in stroke [125]. (**f**) FES study using fNIRS in stroke. Reprinted with permission from Ref. [125]. Copyright 2021, Elsevier. (**g**) MEG-BCI: hand orthosis controlled by ipsilesional central mu-rhythm. Reprinted with permission from Ref. [126]. Copyright 2016, Frontiers.

Electroencephalogram is a widely utilized non-invasive method for monitoring cerebral activity. The method is based on the placement of metal electrodes on the scalp, which are used to measure the small electrical potentials generated outside the head as a result of the action of neurons in the brain. In comparison to other techniques for imaging the brain, EEG has a very high temporal resolution, allowing the tracking of events within the brain with millisecond accuracy. Furthermore, it is in principle portable, thus enabling neuroimaging in real-world settings outside of clinical and laboratory environments. Consequently, it is a highly prevalent sensing method [118,119]. By detecting alterations in these potentials, the data embedded within the EEG signal can be efficiently extracted (Figure 9a), and the patient’s movement intention can be predicted. This has commenced the development of active rehabilitation models for patients with neurological diseases and has been demonstrated to have favorable practical outcomes [120,121]. Closed-loop BCIs that tightly couple movement-related cortical activity with contingent sensory or proprioceptive feedback are explicitly designed to exploit Hebbian and spike-timing-dependent plasticity in sensorimotor networks. In particular, when attempted movement or motor imagery is temporally paired with functional electrical stimulation or other feedback, changes in corticospinal excitability and interhemispheric connectivity consistent with Hebbian plasticity have been demonstrated in people after stroke [122]. These findings support the idea that SSVEP- and MI-based BCIs can drive activity-dependent reorganization by enforcing precise temporal coincidence between intention-related brain activity and peripheral or visual feedback. However, its signal is inadequate, susceptible to artifacts, and has low spatial signal resolution [123], which presents challenges to signal analysis and processing.

Magnetoencephalography employs highly sensitive magnetometers to detect magnetic fields generated by neural currents in the brain. This is predicated on the understanding that the fundamental process of brain activity, namely the propagation of signals between neurons, is dependent on the movement of charged ions. EEG is a technique that measures the electrical potential changes that occur during this activity. As alterations in electrical fields give rise to magnetic fields, MEG is capable of detecting this brain activity with greater spatial resolution than that afforded by EEG. The skull and scalp distort EEG signals, with a minimum spatial resolution of 2–3 cm [127]. In contrast, the head is magnetically transparent, allowing MEG activity maps to have sub-millimeter spatial precision under optimal conditions [128]. Although the remaining principles and analytical methods are consistent with EEG, research on MEG is still in its early stages, and its application in stroke assessment still needs further exploration (Figure 9g). Corsi et al. [129] have combined MEG with EEG and shown good results in MI tasks.

Functional magnetic resonance imaging is a non-radiative technique based on blood oxygenation level dependence (BOLD) to observe brain activity that has been widely used in neuroscience research (Figure 9c) [130]. Among the applications of fMRI, functional connectivity (FC) and effective connectivity (EC) have been demonstrated to be effective methods for evaluating motor rehabilitation status in stroke patients. Ebrahimzadeh et al. [72] review the application of combined EEG and fMRI in the assessment of human brain function. They highlight the complementarity of the two methods in providing high temporal and spatial resolution, as shown in Figure 9b, and explore their clinical and cognitive neuroscience applications, to improve the detection of human brain intentions.

Functional near-infrared spectroscopy is a recently developed brain imaging technique. The fundamental principle underlying fNIRS is analogous to that of fMRI, namely that neural activity in the brain gives rise to alterations in cerebral blood flow [124]. The absorption rates of oxygenated and deoxygenated hemoglobin in brain tissue differ for wavelengths of NIR light between 600 and 900 nm. By Beer-Lambert’s law, these absorption rates can be employed to ascertain brain activity [131]. The technique offers several advantages, including low cost, simplicity, portability, and a compact equipment size. In particular, its capacity for dynamic, real-time measurement without the need for a fixed position offers significant value in tasks that require continuous motion, such as gait control [132]. Furthermore, fNIRS exhibits a superior spatial resolution compared to EEG and MEG. However, its detection depth is limited to approximately 1.5 cm, which restricts its utility to monitoring activity in the outer layers of the brain [133]. Additionally, its temporal resolution is constrained to approximately 0.1 s [134]. Given these attributes, fNIRS has emerged as a valuable tool for investigating neurological disorders such as stroke (Figure 9d–f) [135]. Yang et al. [132] conclude that fNIRS has significant potential in monitoring stroke recovery, evaluating treatment efficacy, and as a research tool. Despite the relatively recent emergence of fNIRS technology, it has already demonstrated particular promise in the detection of neural activity associated with motor tasks [125]. The recently proposed fNIRS-based beam photoelectric method employs 32 photoelectrodes to enhance spatial resolution, with the potential for extension to three-dimensional (3D) fNIRS imaging [136].

In EEG-based motor-imagery BCIs, the most widely used decoding pipelines combine band-pass filtering of sensorimotor rhythms with spatial filtering methods such as common spatial patterns (CSP) or filter-bank CSP, followed by classifiers including linear discriminant analysis and support vector machines. Numerous variants of CSP, including regularized, correlation-based and transformed CSP approaches, have been proposed to improve robustness to noise, small training sets and non-stationarities in MI EEG. Alternative frameworks use covariance-based features and Riemannian-geometry classifiers to exploit the structure of EEG covariance matrices [137]. For fNIRS- and fMRI-driven paradigms, general linear models and block-design analyses are commonly applied to derive hemodynamic activation features from motor-related cortical regions, which are then fed into shallow neural networks or conventional machine-learning classifiers for intention decoding. More recently, convolutional and recurrent neural networks such as ShallowConvNet, DeepConvNet and EEGNet have been developed specifically for EEG, operating on raw time series or time–frequency representations to enable end-to-end learning of motor intention representations across different BCI paradigms [138].

#### 3.1.4. Challenges in Motion Intention Detection

Although diverse intention-detection signals have enabled more intuitive control of active rehabilitation devices, each modality still faces non-trivial limitations when deployed in heterogeneous stroke populations and real-world environments. A critical overarching challenge is the latency–accuracy–reliability trade-off: signals that are closer to the motor cortex can, in principle, detect intention earlier, but usually suffer from lower signal-to-noise ratio and greater sensitivity to artifacts, whereas peripheral status signals are more robust yet intrinsically reactive and strongly influenced by abnormal tone, compensatory synergies and assistive device dynamics. Designers must therefore balance robustness, patient burden, and the depth of neural information captured, while at the same time designing evaluation protocols that fairly compare different strategies across studies and devices.

Somatosensory and biomechanical signals offer a direct window on limb kinematics and interaction forces, but they primarily reflect the consequences of motor commands rather than the underlying motor intent. Recent multimodal sensing work has highlighted that trunk compensation, abnormal synergies and assistive device dynamics can all distort the relationship between measured motion and the patient’s actual voluntary effort, especially in patients with moderate-to-severe paresis. Zhao et al. [18] systematically reviewed sensor configurations and showed that joint angles, surface forces and inertial signals are highly informative for detecting task execution, but less sensitive to “hidden” effort in those who can barely generate overt movement, suggesting a risk of overestimating recovery in patients who rely heavily on compensatory strategies.

Electromyography-based intention detection addresses some of these issues by capturing muscle activation upstream of overt motion, yet introduces its own challenges. EMG signals are non-stationary: electrode shifts, skin impedance changes, evolving spasticity, fatigue-related changes in motor unit recruitment and crosstalk between adjacent muscles produce substantial day-to-day variability in feature distributions, so models trained on one session may degrade quickly when reused without recalibration. Bi et al. [139] showed that even state-of-the-art regression models suffer from limited generalization across sessions and tasks, and that most studies rely on relatively short, well-controlled protocols with small samples of mildly impaired participants. Furthermore, many EMG-based systems still depend on manual threshold tuning or frequent supervised recalibration, which increases the burden on clinicians and constrains use in home-based, long-term rehabilitation. Robust methods for automatic channel selection, adaptive normalization and drift compensation remain an open research need.

Brain-derived signals theoretically provide the most direct access to motor planning and sensorimotor integration, and EEG-based brain–computer interfaces have been extensively explored for post-stroke rehabilitation. However, practical implementation is constrained by low signal-to-noise ratio, inter-individual variability and the phenomenon of “BCI-inefficiency,” where a substantial fraction of users fails to achieve reliable control despite training. Orban et al. [14] reviewed non-invasive BCI systems for rehabilitation and emphasized that EEG features, classifier architectures and feedback protocols must be tuned to individual cortical reorganization patterns, yet most published studies still involve small, fairly homogeneous cohorts and short intervention periods. In addition, the cognitive and attentional demands of EEG-BCI paradigms may be difficult to sustain for patients with co-existing cognitive deficits or fatigue, which raises questions about long-term adherence beyond controlled trials.

Across modalities, there is growing interest in multimodal and hybrid intention-detection strategies that combine complementary sources of information, for example, biomechanical sensors with EMG, or EMG with EEG, to improve robustness and provide richer context about both movement execution and motor intent. Such fusion can attenuate the weaknesses of individual signals and enable more adaptive, user-specific control policies, but also increases system complexity, costs and the risk of overfitting to narrowly defined experimental conditions. A scoping review of intention detection strategies for upper-limb orthoses further highlighted that evaluation protocols and usability metrics remain heterogeneous, limiting rigorous comparison of different modalities and fusion schemes in terms of real-world performance, user acceptance and suitability for daily life contexts. Future work therefore needs not only better algorithms, but also standardized, ecologically valid benchmarks that capture how well intention-detection systems support sustained, meaningful practice across the spectrum of stroke severity.

### 3.2. Techniques and Devices for Feedback

The extant classification standards for intervention methods predominantly concentrate on disparate body regions that are the recipients of interventions (such as the brain [140,141], UE [142,143], and LE [144,145]) or on distinct types of intervention signals [72,146]. The former provides a foundation for physicians to select corresponding rehabilitation intervention methods based on the area of movement impairment in stroke patients. The latter is oriented towards the classification of signal types and the application of unique features, as well as the analysis and processing algorithms that are involved in the intervention devices of different principles.

Although the aforementioned two classification approaches offer a relatively systematic overview of existing technological pathways and rehabilitation methods, both are deficient in fully considering the significant impact of patients’ subjective emotional experiences on the rehabilitation process. Research has indicated that the self-control level of patients during rehabilitation is intricately linked to the recovery and improvement of post-stroke functions. There is a positive correlation between the maintenance of a positive emotional state and superior rehabilitation outcomes. Conversely, negative emotions such as depression exhibit a negative correlation with the enhancement of functional abilities and motor skills [147].

In the Section 3.2, we organize feedback interventions according to the primary site of intervention and the modality of stimulation. First, we distinguish interventions according to their main site of action into three families: (i) task- and environment-based sensory feedback systems, which act primarily at the level of sensorimotor interaction and cognitive–emotional engagement; (ii) non-invasive central and peripheral neuromodulation techniques, which deliver electrical, magnetic or ultrasonic stimulation directly to neural tissue; and (iii) closed-loop central–peripheral feedback systems based on neural priming, in which neuromodulation is systematically coupled with task-specific training and real-time sensory feedback. Within each family, we then specify the dominant modality of stimulation (electrical, magnetic, robotic/mechanical, sensory or multimodal) to highlight practical choices for clinical implementation. From the patient’s perspective, these groups also differ in the directness and intensity with which the intervention is perceived—from largely “non-contact” auditory and visual experiences to physically felt electrical or mechanical stimulation and deeply immersive closed-loop training—which can influence motivation and emotional responses. The core technologies, clinical manifestations and main results of these feedback methods in stroke rehabilitation are summarized in Table 2.

#### 3.2.1. Task-Level Sensory Feedback

**Figure 10:**
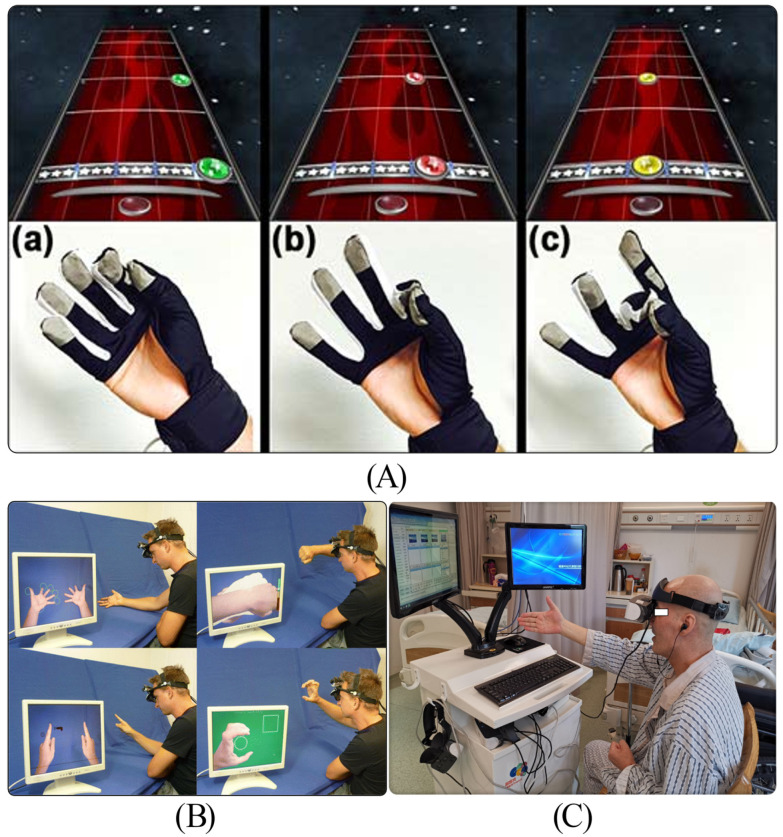
(**A**) MusicGlove device used in the study. Users can obtain visual cues by scrolling notes on screen (top), enabling them to promptly grasp the specific finger positions for popular songs. (Grips include: (a) key pinch grip; (b) pincer grip; (c) finger-thumb opposition with second, third, and fourth fingers). Reprinted with permission from Ref. [149]. Copyright 2016, eScholarship. (**B**) An AR home-training system. Reprinted with permission from Ref. [150]. Copyright 2014, Springer. (**C**) The Mirror Neuron System Training (MNST) machine and patient training status. Reprinted with permission from Ref. [151]. Copyright 2020, Wiley.

Within task- and environment-based sensory systems, the current post-stroke intervention methods are primarily based on auditory [148] and visual therapies (Figure 10). From the perspective of stimulation modality, these task-level interventions therefore mainly deliver auditory and visual sensory cues, rather than electrical or magnetic currents applied directly to neural tissue.

In the initial phases of stroke recovery [152], musical listening has been demonstrated to facilitate relaxation, enhance mood, and promote physical and mental activation. Music-based interventions have been shown to play a role in alleviating anxiety in post-stroke rehabilitation patients [153]. “Music-supported therapy” (MST) engages patients in music composition through activities such as playing simple melodies on a drum pad or piano keyboard, thereby establishing feedback on how sound correlates with movement execution [154]. Rhythmic Auditory Stimulation (RAS) is also widely utilized in UE and LE movement cues.

Mirror therapy is a form of MI training based on repetitive imagination and psychological exercises. It utilizes a mirror apparatus to replicate the movements of the unaffected limb onto the affected side. Patients engage in movement observation, imitation, and relearning through visual feedback. By consistently stimulating the motor cortex of the brain through visual feedback, mirror therapy influences cortical electrical activity and excitability, thereby fostering brain functional reshaping and inducing the recovery of motor functions.

**Table 2:** Engineering technology and clinical performance of different feedback methods.

Studies	Participants Recovery Phase	Feedback Targets	Feedback Modalities	Duration of Intervention	Contributions	Main Results
	Task-Level Sensory Feedback					
[149]	17 Cp *	Hands	Music Glove	3 W *, 9 T *, 9 H *	The MusicGlove has been demonstrated to be a feasible and effective home therapy that motivates users to complete a large number of therapeutic grasping movements.	The MusicGlove group exhibits greater improvements in Motor Activity Log Quality of Movement and Amount of Use scores (*p* = 0.007 and *p* = 0.04, respectively).
[150]	7 Hp *	Hands	Mirror and imagery treatment	10 T *, 2.5 H *	An AR-based home training system is developed to treat phantom limb pain, complex regional pain syndrome (CRPS), and impaired motor control after stroke.	Performance on four tasks improves significantly, and the system can help keep patients motivated when used over longer periods.
[151]	60 Cp *	UE *	Mirror neuron system (MNS)-based training	8 W *, 40 T *, 14 H *	This is the first report on the effectiveness of MNS on both motor and cognition function in a cohort of stroke patients over a relatively long period.	The MNS group shows improved UE motor function and cognitive function (*p* < 0.05).
[153]	44 Ap *	Mental health	Music Rhythm	1 H *	Music intervention may help lessen anxiety in rehabilitation patients poststroke.	Participants report significantly less anxiety (*p* < 0.0001) compared to before the intervention.
[155]	13 Cp *	Trunk and UE *	Music Upper Limb Therapy-Integrated (MULT-I)	6 W *, 12 T *, 9 H *	MULT-I helps stroke survivors rebuild their sense of self by integrating sensorimotor, affective, and interoceptive information.	FM scores increase significantly (*p* = 0.007), and this intervention may be more effective in a subgroup of patients with lower function.
**Non-Invasive Neuromodulation**						
[156]	24 Cp *	UE *	High-definition transcranial electrical theta burst to superimpose direct current stimulation (HD-tDCS-eTBS)	4 W *, 12 T *, 10 H *	Accompanied with conventional rehabilitation, HD-tDCS-eTBS significantly reduce UE spasticity.	Shoulder adductors improve by 38.5% and elbow extensors improve by 61.5%.
[157]	34 Sp *	Memory	tDCS + rTMS	4 W *, 20 T *, 7 H *	Demonstrating the effectiveness of rTMS-tDCS bimodal stimulation in treating patients with post-stroke amnesia.	The total scores of the four clinical scales are significantly improved (*p* < 0.05).
**Closed-Loop Feedback Systems**						
[158]	30 Cp *	Hands	SSVEP-BCI-controlled soft robotic glove	2 W *, 10 T *, 10 H *	The effect of rehabilitation is better than that of the robotic glove alone, proving the feasibility of SSVEP-BCI-controlled soft robotic glove in hand function rehabilitation.	FMA-UE * increases by 10.5 ± 8.05 between rehabilitation.
[159]	N/A *	Ankle joint	S-ARR	N/A *	Designed for early bedridden rehabilitation.	Joint angles < 2°
[160]	33 Sp *	UE *	BCI + FES	4 W *, 18 T *, 12 H *	Facilitating durable motor recovery in patients with low BCI performance.	FMA-UE * increases by 89.7%.
[161]	1 Hp *	Ankle joint	Ankle rehabilitation robot	N/A *	Design of a bilateral ankle rehabilitation robot that supports three degrees of freedom.	Maximum position error < 5%.
[162]	10 Hp *	UE *	A wearable supernumerary robotic limb (SRL) system	N/A *	The SRL system can effectively assist patients with UE movement disorders to perform UE tasks in daily life through natural human–computer interaction.	The MI classification accuracy is effectively improved (90.04%), and all subjects can complete the target object grasping task within 23 s, with the highest success rate reaching 90.67%.
[163]	N/A *	Gait	VR-based treadmill train	N/A *	Virtual setting with gait training tasks and real-time feedback.	The designed exergame supports gait rehabilitation and has the potential to be used in TR training environments.
[164]	11 patients	Hands	VR	N/A *	Adaptive sports games based on VR and cluster analysis.	SUS scores are all above 60 (full score is 100).
[165]	6 patients and 6 Hp *	Grip strength	VR	3 W *, 6 T *, 3 H *	Increased UE * muscle activation can be measured through physiological indicators (skin electrodermal activity).	The system helps increase grip strength (one subject increased grip strength by 2 kg), aiding functional recovery.
[166]	12 Hp *	LE	An enhanced MI-BCI based on FES and VR scenario	N/A *	It is verified that the use of the proposed MI-BCI could improve the classification accuracy and motor cortex activation in subjects by visual guidance and FES.	The classification performance is significantly improved (*p* < 0.05) by using the FES + VR paradigm and the activation intensity of the motor cortex based on the FES + VR paradigm is higher than that based on the VR paradigm, especially at channels C3 and Cz.
[167]	5 Hp *	Gait	BCI + VR	N/A *	Visual feedback provided by VR technology is used to enhance the performance of BCI in MI tasks and accurately distinguish the user’s MI state from the rest state.	The accuracy of issued commands is 91.0 ± 6.7.

* Hp—Healthy person(s), Ap—Acute phase, Sp—Subacute phase, Cp—Chronic phase, N/A—not available, W—week(s), T—time(s), H—hour(s), FM—Fugl-Meyer, FMA-UE—Fugl-Meyer Motor Assessment of Upper Extremity, SUS—System Usability Scale.

#### 3.2.2. Non-Invasive Neuromodulation

Non-invasive feedback technologies can be classified according to the level of neural system stimulation into two main categories: peripheral stimulation techniques and central stimulation techniques [168]. The most commonly employed non-invasive peripheral stimulation techniques include FES, sensory stimulation, muscle electrical stimulation, and TES. The combination of central and peripheral interventions is likely to result in the formation of a closed-loop feedback system [169], which has the potential to enhance brain plasticity and facilitate neural pathway remodeling. Consequently, in recent years, NIBS and spinal cord stimulation techniques, including but not limited to TMS, tDCS, transcutaneous spinal cord stimulation (tSCS), and transcranial-focused ultrasound (TUS), have been introduced to achieve combined rehabilitation interventions involving peripheral and central stimulation. Of these, NIBS and NMES are the most commonly employed.

Non-invasive brain stimulation techniques encompass two primary modalities: TMS and TES [170]. Within these categories, different variants have demonstrated efficacy in stroke patients, including rTMS, continuous theta burst stimulation (cTBS), intermittent theta burst stimulation (iTBS), tDCS, tRNS, and tACS. Additionally, combined therapies can be employed, including combinations of electrical and magnetic stimulation protocols or the combination of brain stimulation with peripheral stimulation.

Neuromuscular electrical stimulation employs brief external electrical pulses to stimulate peripheral nerves by modulating neuronal depolarization or hyperpolarization [72]. NMES induces muscle contractions through surface electrodes on the skin, transcutaneous electrodes, or implanted electrodes. Typical NMES parameters include pulse frequency (10~100 Hz), amplitude (10~120 ms), and pulse width (200 μs~1 ms). Higher-frequency NMES generates greater force but results in muscle fatigue and a rapid decline in contraction strength. Broader pulse widths result in more pronounced cortical and muscle responses.

#### 3.2.3. Closed-Loop Feedback Systems

In closed-loop central-peripheral feedback systems based on neural priming, the activation of the motor cortex is associated with changes in neural plasticity [171], which has positive implications for the improvement of motor function in patients who have suffered a stroke [172]. Among the various neural activation mechanisms, those that are initiated based on actual movement and MI are the most widely applied.

**Figure 11:**
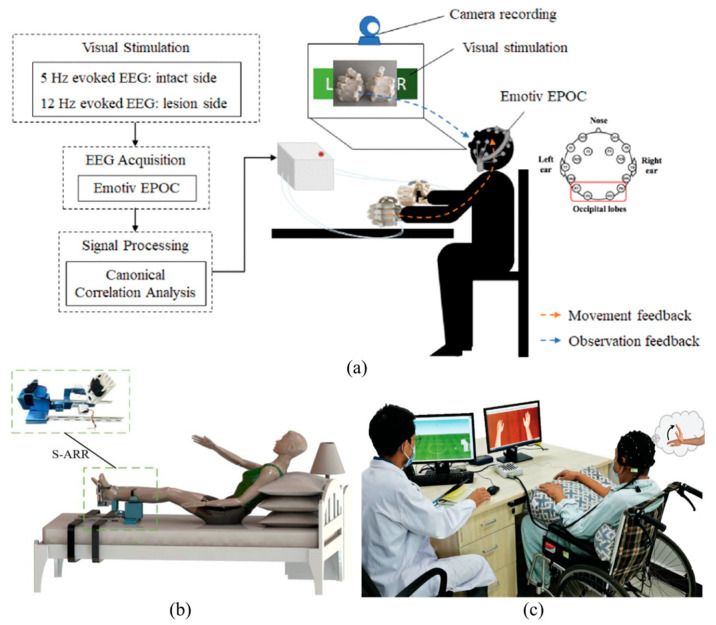
(**a**) Schematic diagram of the steady state visually evoked potential (SSVEP)-BCI system. Reprinted with permission from Ref. [158]. Copyright 2022, IEEE. (**b**) Three-dimensional model of the patient using a Supine ankle rehabilitation robot (S-ARR). Reprinted with permission from Ref. [159]. Copyright 2023, MDPI. (**c**) BCI-based rehabilitation treatment). Reprinted with permission from Ref. [160]. Copyright 2023, IEEE.

The topic of RAT is currently a focus of considerable research interest, largely due to its potential applications in understanding the mechanisms underlying motor initiation. Exoskeletons or mechanical arms can be employed to restore LE gait [173] and UE motor functions, respectively. These devices facilitate movement along different axes, thereby providing enhanced control and monitoring capabilities for specific tasks and patient needs [82,174,175,176,177], as shown in Figure 11b. It is important to note that the impact of RAT is contingent upon many factors, including the type of support provided for the limbs, the patient’s ability to perform movements, the duration of robot support, and the type of movements. Furthermore, research on the effects of using only robots for therapy is limited. However, combining RAT with other techniques to improve overall motor and cognitive function in patients yields more significant results [178].

The advancement of BCI technology offers a viable method for passively identifying subjective MI in patients (Figure 11a,c), while VR technology has emerged as an optimal approach for actively directing patients to generate predefined MI.

A brain–computer interface is a technology that translates the electrical, magnetic, or metabolic activity of the brain into control signals for external devices [179]. Such devices have the potential to replace, restore, enhance, supplement, or improve natural neural output, thereby altering the interaction between the brain and its external or internal environment. Under the level of invasiveness, BCIs can be classified into two principal categories: non-invasive (extracranial) and invasive. In invasive systems, electrodes are positioned on the surface of the brain (ECoG) or implanted within the cortex (microelectrode arrays). In non-invasive systems, electrodes are positioned on the scalp, with the most common techniques being EEG and NIRS. In typical EEG-based non-invasive BCIs, the real-time decoding of a user’s motor intentions (MI or execution) from ongoing brain electrical activity is achieved through the extraction of relevant features. In typical experiments, the detection of motor intentions results in the generation of sensory feedback for the user. The feedback can be in the form of abstract stimuli, such as a moving cursor on a computer screen, or concrete stimuli, such as a visual representation of the participant’s body parts overlaid on a virtual image on a computer screen or directly overlaid on the participant’s The limbs may be represented in a VR head-mounted display, or they may be provided through somatosensory representations via robots, tactile or NMES systems. This allows the reproduction of the intended movements, which has been demonstrated to enhance motor function in patients.

VR therapy entails the utilization of computer interfaces to simulate interactions between patients and their surrounding environment, employing a combination of hardware and software. This enables the formation of sensory connections that are closely aligned with reality, while also offering the additional advantage of undertaking tasks in real-time with immediate feedback [180]. VR systems are designed with the specific objective of facilitating the rehabilitation of patients with neurocognitive disorders. The customizable interfaces are intended to cultivate functional skills that can be applied in the real world [181,182]. The objective of neurorehabilitation is to facilitate intensive repetitive movements [183], comprising simple actions, to improve motor function. Furthermore, neurorehabilitation is often associated with a lack of feedback and monotonous autonomous home exercises, which can result in a decline in patient motivation and adverse effects on emotional states due to the intensity of the therapy. In response to this, the introduction of VR and AR technologies can simulate real-life scenarios, thereby enhancing the enjoyment of rehabilitation training and boosting patient willingness to recover. To facilitate a more seamless transition to post-rehabilitation life, VR technology enables the integration of the home environment into the hospital setting for training prior to discharge [184]. Furthermore, in order to further enhance the portability of treatment equipment, Song et al. have developed motion games for UE motor function and cognitive training on mobile phones (portable devices). Patients are able to touch virtual targets generated in a 3D AR environment by moving their arms. Following the successful touching of each target, patients receive visual and vibrational feedback. The performance of motor functions is assessed based on performance in AR games, and various aspects of mental state are evaluated through questionnaires for self-reported mental status [185]. From a mechanistic standpoint, VR- and game-based programs combine high-dose, task-specific practice with enriched multisensory and motivational environments. Experimental and clinical studies have shown that enriched environments and intensive motor training can upregulate BDNF and related neuroplasticity markers and can accelerate functional recovery after cerebral ischemia and other central nervous system injuries [186,187]. Therefore, VR-based rehabilitation leverages these activity- and reward-dependent pathways by providing a structured form of enriched, task-oriented practice. At the same time, most current stroke VR trials have not yet directly measured BDNF levels, myelin changes or other molecular markers, so we treat these links as a mechanistic rationale grounded in broader neuroplasticity research rather than as fully established molecule-specific effects of individual VR systems.

Beyond motor rehabilitation, BCI-based neuroprostheses are increasingly being explored for restoring or augmenting sensory and higher cognitive functions. In the visual domain, ocular neuroprostheses such as retinal and cortical implants seek to provide interpretable visual percepts in blind or severely visually impaired individuals by electrically stimulating surviving retinal cells or visual cortex populations and decoding neural responses to build functionally useful representations of the environment. Recent reviews have summarized how retinal implants, optic nerve and cortical visual prostheses are moving toward higher resolution, improved biocompatibility and integration with real-time image processing [188,189,190]. Auditory neuroprostheses offer another mature example of activity-dependent plasticity being harnessed for sensory restoration. Cochlear implants, which bypass damaged hair cells and directly stimulate spiral ganglion neurons, have become a highly successful neural prosthetic for restoring hearing in patients with severe to profound deafness. Rudroff et al. [191] have proposed AI- and BCI-enhanced communication neuroprosthetic frameworks to jointly address speech production and auditory processing impairments by combining non-invasive brain stimulation, neural decoding and intelligent signal processing. Similar principles are being extended to somatosensory and even olfactory systems. Neuromorphic hardware and closed-loop stimulation paradigms for somatosensory neuroprostheses aim to deliver biomimetic tactile feedback by interfacing with peripheral nerves or somatosensory cortex, thereby supporting more natural interaction with prosthetic limbs [192]. Morozova et al. [193] demonstrated that frontal theta rhythms can be used as a neural signature in an EEG-based olfactory BCI. In future work, similar concepts of intention detection, neural priming and closed-loop feedback may be adapted to address post-stroke sensory, communication and cognitive deficits [194], thereby extending active rehabilitation paradigms beyond the motor domain. In terms of stimulation modality, such closed-loop systems typically combine robotic or mechanical actuation with multimodal sensory feedback (visual, auditory and proprioceptive), and in some protocols concurrent electrical neuromodulation.

#### 3.2.4. Challenges in Feedback Techniques and Devices

As shown in Table 2, task-level sensory feedback approaches—such as mirror therapy, action observation, music-based interventions and VR-enhanced training—are supported by a relatively large number of small-to-moderate sized trials, often in subacute and chronic patients with mild-to-moderate impairment. These interventions, which are experienced by patients as largely “non-contact”, consistently improve motor scores and activities of daily living when embedded in task-specific practice, but effect sizes are generally modest and long-term follow-up data are limited. Non-invasive neuromodulation techniques, including tDCS, rTMS and peripheral NMES/FES, have been evaluated in a growing number of randomized or controlled studies and can produce measurable gains in motor function and sometimes cognition; however, their impact is highly sensitive to stimulation parameters, target selection and timing relative to training [195,196], and inter-individual variability remains substantial. Closed-loop feedback systems based on neural priming—such as EEG- or fNIRS-driven BCIs combined with FES or robotic devices—represent a deeper level of central–peripheral engagement and are particularly attractive for patients with more severe impairment and limited voluntary movement. At present, most of these studies are proof-of-concept trials with small, heterogeneous cohorts and technically complex protocols, which show promising improvements in upper-limb function and engagement but do not yet allow firm conclusions about comparative effectiveness or scalability. In Table 2, closed-loop BCI and hybrid VR–FES prototypes typically enroll fewer than 20 participants per study, whereas task-level sensory feedback and non-invasive neuromodulation are increasingly evaluated in randomized or controlled trials with sample sizes between about 30 and 160 stroke survivors. Overall, Table 2 indicates that task-level sensory feedback and non-invasive neuromodulation currently provide the strongest evidence base for broader implementation, whereas closed-loop, deeply engaging feedback systems remain a high-potential but still exploratory direction that requires larger, pragmatic trials.

In addition, several practical challenges still limit the translation of these feedback families from proof-of-concept studies to scalable clinical practice. For task-level sensory feedback, the therapeutic effect depends not only on the physical dosage of practice, but also on patients’ cognitive reserve, emotional state, and sensory preferences. For non-invasive neuromodulation, the central difficulty lies in navigating a high-dimensional parameter space while maintaining clinical robustness and patient acceptability. Peripheral NMES/FES has more direct and intuitive effects on movement, spasticity and gait, and meta-analyses support its ability to improve activity when combined with conventional rehabilitation, but they also underline discomfort, rapid muscle fatigue, skin irritation and suboptimal long-term adherence as persistent barriers to widespread use [197,198]. The observations point to the need for adaptive, fatigue-aware parameter tuning, more ergonomic electrode and device designs, and pragmatic trials that explicitly report tolerability and adherence rather than focusing solely on impairment-level outcomes. Closed-loop feedback systems suggest that coupling attempted movement or motor imagery with real-time visual or proprioceptive feedback can enhance the sense of agency and support motor gains, particularly in patients with severe impairment [199]. Immersive environments can enhance motivation and engagement, but they may also provoke cybersickness, attentional overload or safety issues in frail or cognitively impaired patients, and existing trials rarely stratify or adapt VR content based on neuropsychological profiles. A key unresolved problem is how to systematically personalize task difficulty, sensory complexity and narrative content to match individual cognitive and emotional characteristics, rather than relying solely on therapist-driven, session-by-session adjustments.

Across the three feedback families, a common unmet need is the integration of technical performance metrics with patient-centered outcomes and real-world feasibility. Most studies involve small samples, short intervention periods and heterogeneous outcome measures, with limited reporting of adherence, usability, emotional impact or long-term participation in meaningful daily activities. Future research should prioritize multi-arm, adequately powered clinical trials that compare different feedback intensities and modalities, incorporate neurophysiological, behavioral and affective outcomes within the same framework, and explicitly address deployment in telerehabilitation and smart-home environments. Only by tackling these translational challenges can sophisticated feedback technologies be embedded into personalized, sustainable active rehabilitation pathways for diverse stroke populations.

In this section, the mechanism of action of neuroplasticity in active rehabilitation is used as the basis for dividing active rehabilitation technology for stroke patients into two distinct aspects: detection technology and feedback technology, which provides a reference for doctors to choose appropriate rehabilitation intervention methods based on the patient’s condition by offering a range of intention detection methods.

## 4. Treatment Options Designed for Active Rehabilitation

Numerous studies have systematically reviewed the efficacy of different detection and feedback methods in the active rehabilitation process. However, the majority of individuals tend to overlook the reality that patients at varying stages of rehabilitation frequently possess disparate rehabilitation requirements. This oversight may result in misguided judgments by medical professionals when formulating rehabilitation plans for patients, ultimately leading to suboptimal treatment outcomes. Oyake et al. [200] find that 75% of medical staff decided which motivational strategy to use (which can be learned through clinical experience) by considering comprehensive information about the patient’s health status, environmental factors, and personal factors. This approach focuses on the patient’s subjective feelings while ignoring the objective impact of the hardware system used in the rehabilitation process. Yoshida et al. [201] underscored the significance of interventions tailored to external factors in maintaining patient motivation. Clinical assessment based solely on observation of patient behavior is susceptible to errors. Rehabilitation professionals are therefore advised to utilize validated assessment scales for comprehensive evaluation. Consequently, the design of a personalized rehabilitation program for the patient represents a pivotal aspect of the entire treatment process.

The rehabilitation of patients who have suffered a stroke is frequently divided into four distinct phases: the hyperacute, acute, subacute, and chronic phases. During the hyperacute and acute phases, the patient’s neurological damage is still evolving. At this juncture, it is inadvisable to introduce rehabilitative intervention, as this could potentially exacerbate the damage already incurred. In the subacute phase of stroke, the window of neuroplasticity opens, rendering this a crucial period for stroke recovery. This concept is analogous to the phenomenon of enhanced plasticity during development, which is referred to as the critical period. In the chronic phase, the plasticity window will gradually diminish and will no longer be capable of spontaneous recovery. Consequently, intensive neuro- rehabilitation treatment is necessary to achieve limited results. Active rehabilitation is primarily employed as an enhanced neurotherapy method in the subacute and chronic phases to intervene in the post-stroke rehabilitation process.

To furnish medical practitioners with a more comprehensive and objective reference for the design of rehabilitation programs, this section presents an overview of the therapeutic effects of different rehabilitation programs at different stages of the rehabilitation process and provides a detailed account of the design principles underlying each program. Most of the protocols and technologies described here are intended for patients who are at least able to follow simple commands and participate, to some extent, in task-oriented training. At the same time, it is important to acknowledge that a substantial proportion of patients in the early phases present with disorders of consciousness (DoC), such as coma, unresponsive wakefulness syndrome/vegetative state (UWS/VS), minimally conscious state (MCS) [202] or locked-in syndrome (LIS). These conditions require adapted goals and rehabilitation strategies, which differ from those applied to patients who can actively engage in therapy and are briefly outlined in the acute-phase Section 4.1 below. Based on a synthesis of clinical evaluation and engineering design, we re-examine the active rehabilitation measures and research that can be employed at different stages following a stroke. To interpret the strength of clinical evidence across phases and technologies, we qualitatively distinguish between different levels of support. Single-case reports, feasibility studies in healthy volunteers and small pilot trials (typically involving fewer than about 15 participants) are cited as proof-of-concept demonstrations of emerging approaches. Larger randomized or controlled clinical trials and systematic reviews or meta-analyses—often including tens to hundreds of stroke survivors—carry greater weight when evaluating effectiveness and drawing overall conclusions. We therefore report sample size, study population and, where applicable, trial design, and contrast exploratory versus clinically validated interventions.

### 4.1. Acute Phase

Patients in the acute phase, defined as the initial seven-day period following a stroke, often exhibit a lack of motor function. During this stage, the primary objectives are to prevent further brain damage and to facilitate the gradual restoration of cognitive abilities. The objective of acute phase rehabilitation is to stabilize the condition, reduce complications, and establish a foundation for subsequent rehabilitation. During this period, moderate rehabilitation intervention can markedly enhance the patient’s capacity to perform daily living activities. However, excessive rehabilitation intervention carries inherent risks and may precipitate complications such as thrombosis, inflammatory infection, and dysphagia. Consequently, the rehabilitation plan must be tailored to the individual and strike a balance between the intensity of intervention and the patient’s tolerance. Such considerations include the rehabilitation environment, training standards, and other neurostimulation -based treatment approaches.

Approximately 90% of patients with rehabilitation needs elect to pursue home-based treatment [203]. Consequently, the rehabilitation and care provided in this setting must be supported by reasonable rehabilitation plans. The collaborative care model (CCM) is initially proposed by Lott in 1992, based on Orem’s self-care theory [204]. It employs a limited set of human and material resources to facilitate patient and family involvement in the care of patients, enable patients to fully utilize their self-care capabilities, and enhance the quality of life for patients and their families. The model emphasizes coordination and continuity of care, ultimately leading to improved overall care. Wu et al. [205] conduct remote rehabilitation exercise training based on a collaborative care model for patients’ limb coordination, gait balance, and joints. The results demonstrate that the intervention group exhibited greater improvement in Fugl-Meyer (FM) motor function, Berg Balance Scale (BBS), and stroke-specific quality of life scale assessment than the control group. This indicates that the Internet-based remote rehabilitation model has superior rehabilitation continuity. Early rehabilitation measures effectively ensure the joint activities of patients and establish a foundation for subsequent recovery training.

In the acute phase of post-stroke recovery, repetitive, high-dose, task-specific training has been demonstrated to enhance beneficial neuroplasticity [206]. However, physical therapists may not always be able to achieve optimal training standards. Consequently, for patients with high training standards, the use of machine assistance is a viable option. For example, Nolan et al. [207] conduct robotic exoskeleton gait training to provide patients with a high-step dose and task-specific movements. The experimental group, which receive robotic assistance, walk a distance that is twice as great as that walked by the control group over the same training duration. Furthermore, the Functional Independence Measure (FIM) assessment demonstrates a higher score for the experimental group than for the control group. Park et al. [208] design a game-based VR rehabilitation program for UE movement using a glove-type wearable device and examine factors such as multi-lateral hand function. Significant improvements are observed in the hand strength test, the Jebsen-Taylor hand function test (JTHFT), and the Korean version of the modified Barthel Index (K-MBI) of 22 patients (*p* < 0.05). The device is a lightweight, high-precision wearable device that can be worn directly on the patient’s paretic hand, providing visual and auditory feedback simultaneously and inducing the movements necessary for daily activities. This device differs from those used in previous studies in that it allows subjects to use their hands more diversely. Consequently, rehabilitation programs employing wearable devices may facilitate greater functional recovery [209], which may be attributed to the patient’s heightened interest and immersion during training.

Other adjunctive neurostimulation approaches have been demonstrated to diminish infarct size and mitigate post-stroke neurological impairment by attenuating inflammation, safeguarding neurons in the ischemic region, and stimulating angiogenesis and neurogenesis. Transcranial direct current stimulation has been shown to enhance interhemispheric inhibition (unrestricted inhibition from the healthy hemisphere impedes the lesion side) by modulating local cortical excitability [210] (either by increasing excitability on the lesion side, reducing inhibition from the healthy side, or a combination of both) [211]. Bornheim et al. [212] focus on the effects of tDCS on acute stroke patients and involve follow-up observations for up to one year. Their findings indicate that all outcomes of functional movement demonstrated statistically and clinically significant improvements (*p* = 0.02), thereby demonstrating that the application of tDCS during the acute phase of stroke can not only accelerate functional recovery but also enhance it, with the effect being maintained for up to one year after the stroke. VNS represents an emerging adjunctive therapy, encompassing both invasive and non-invasive VNS techniques. Clinical studies have demonstrated that invasive VNS in conjunction with rehabilitative therapy can effectively enhance UE mobility and cognitive function in patients who have experienced a stroke. Non-invasive vagus nerve stimulation, including techniques such as ear and neck vagus nerve stimulation, can also stimulate the vagus nerve to project to the central nervous system, thereby producing effects that are analogous to those observed with invasive vagus nerve stimulation, albeit with a reduced incidence of adverse effects and complications. Berthon et al. [213] demonstrate that fiber activity induced during VNS can be utilized as a biomarker to personalize the dose of VNS. This personalized dose control can enhance the precision, safety, and efficacy of VNS therapy, ensuring that each patient receives a treatment plan that optimally aligns with their unique needs. Furthermore, Zhang et al. [214] present a study on the utilization of rTMS to regulate the cervical vagus nerve in patients with traumatic brain injury (TBI) and cognitive impairment. This study provides preliminary evidence of the potential for rTMS to be applied to the vagus nerve in clinical practice, thereby offering a novel avenue for acute rehabilitation following a stroke.

In addition, in the hyperacute and acute phases, a subset of patients present with severe impairment of consciousness, including coma, UWS/VS, MCS and LIS, often as a consequence of large hemispheric infarction, brainstem stroke or global hypoxic–ischemic injury. For these patients, rehabilitation priorities differ from those of fully conscious stroke survivors and primarily involve optimizing medical stability, preventing secondary complications (e.g., contractures, pressure injuries and infections), promoting arousal and awareness, and establishing basic communication whenever possible. Recovery from DoC is a dynamic process that can extend over months, and that patients with MCS have a better prognosis than those in VS/UWS, reinforcing the need for ongoing assessment and appropriately adapted rehabilitation goals [215,216,217]. A range of interventions have been explored to facilitate arousal and responsiveness in patients with DoC, although much of the evidence comes from traumatic rather than purely ischemic or hemorrhagic etiologies. Multimodal sensory stimulation programs, which deliver structured auditory, tactile, visual, olfactory and proprioceptive stimuli, aim to increase arousal and elicit purposeful behaviors. Norwood et al. [218] found that multimodal sensory therapy may facilitate arousal in minimally conscious or comatose adults with acquired brain injury. Gatling et al. [219] showed that structured sensory stimulation in the acute setting may improve consciousness in children with DoC. In parallel, EEG- and fMRI-based BCIs are being developed as diagnostic and communication tools for patients with DoC. Huang et al. [220] developed a hybrid asynchronous BCI enabling yes–no communication in patients with VS and MCS. From the perspective of post-stroke active rehabilitation, these interventions highlight that even in patients with severely impaired consciousness, activity-dependent plasticity may still be harnessed, albeit with different targets and expectations. Rather than aiming for full independence, the realistic goal for many patients with DoC is to maximize arousal, facilitate basic communication, support participation in simple interactions and optimize comfort and medical stability. When recovery progresses and patients transition from DoC to states in which they can follow commands and engage in task-oriented activities, the phase-specific active rehabilitation strategies described in the subsequent subsections can be gradually introduced and individualized according to the patient’s maximal attainable functional state.

In summary, studies in the hyperacute and acute phases support early but carefully titrated rehabilitation that balances the potential benefits of activity-dependent plasticity against the risks associated with medical instability. For patients who are able to follow simple commands, moderate, structured task-specific training—potentially augmented by robotic assistance, wearable or game-based systems and carefully dosed non-invasive neuromodulation—appears to improve motor function and activities of daily living when delivered under appropriate monitoring. At the same time, most trials are single-center studies with relatively small samples, heterogeneous protocols and short follow-up, and very early, high-dose mobilization remains controversial: the AVERT trial, for example, reported that extremely early, intensive out-of-bed activity (within 24 h of stroke onset) was associated with worse functional outcomes compared with standard care, highlighting the importance of carefully controlling the timing and intensity of early mobilization [221]. For patients with disorders of consciousness, multimodal sensory stimulation programs and emerging EEG- or fMRI-based BCI paradigms may facilitate arousal and basic interaction, but these approaches are still at an exploratory stage and require cautious interpretation.

### 4.2. Subacute Phase

Following the onset of the subacute phase, the patient’s inflammatory response declines, while functional neurons, peri-infarct tissues, and their connection areas undergo rapid growth, and neural plasticity enters the optimal window of recovery. To fully leverage the beneficial effects of rehabilitation therapy in facilitating early functional recovery of stroke patients within a three-month timeframe, active intervention strategies employed during the subacute rehabilitation phase often exhibit characteristics such as high intervention intensity, deep feedback stimulation, and robust functional targeting, which includes techniques such as NES, BCIs based on MI, RAT, and rTMS. Furthermore, many engineering design and clinical evaluation studies have been conducted to address different patient rehabilitation conditions, movement strategies, functional recovery, and postural control and balance needs.

#### 4.2.1. Demand for Rehabilitation Conditions in Subacute Phase

Depending on the severity of stroke in different patients, training programs need to be designed taking into account the requirements of rehabilitation conditions. One is the need for home or ‘one-to-many’ therapy, which is particularly important in countries and regions where there is a shortage of physiotherapists or occupational therapists. The second is the need for high-intensity and high-quality exercise during the critical recovery period, which can be effective in promoting rapid recovery in the post-stroke period.

The design of economically sustainable rehabilitation programs is critical for future household penetration and mass adoption. In a single-center trial involving 111 subacute patients, Aprile et al. [222] evaluate the efficacy of robotic UE therapy using a set of 4 robots and sensor-based devices and develop a new organizational model of 1 physiotherapist supervising 3 subjects, which find significant improvements in UE motor function, activity, and participation using Fugl-Meyer assessments (FMA) before and after treatment. This protocol provides similar treatment times to conventional therapy but reduces staffing requirements, supporting its clinical utility and efficacy. Rémy-Néris et al. [223] use a game-based gravity-assisted therapy system to help patients with moderate to severe movement disorders to perform independent exercises of the UE, thus providing an additional simple method of treatment to increase treatment time during the critical recovery period.

High-intensity, high-dose therapy that focuses on the quality of movement can help to partially restore the patient’s active control of movement in the subacute phase after stroke, rather than just compensatory movement. An immersive animation experience combined with weight support may provide a more effective, enjoyable, and scalable treatment modality for high-dose UE rehabilitation. Krakauer et al. [224] deliver high-dose, high-intensity, motion-quality focused therapy using a novel exploratory neuroanimation therapy (NAT) and find that the Action Research Arm Test (ARAT) improves significantly, although FM-UE scores do not. Robotic devices can control and quantify the intensity of motor tasks, measure and control kinematics and kinetics, and provide repetitive, adaptive, and reinforcing treatments. In a single-center randomized trial with 53 participants, Franceschini et al. [225] perform a goal-directed planar stretching task using a planar end-effector robot for 6 weeks, with a significant improvement in Fugl-Meyer Motor Assessment of Upper Extremity (FMA-UE) (*p* < 0.001), and this improvement is maintained during the 6-month follow-up period.

#### 4.2.2. Strategies to Improve Motor Learning

In the aftermath of a stroke that results in activity limitation and disability, it is estimated that between 80 and 90% of patients will develop paralysis, which can lead to severe injury, loss of activities of daily living (ADLs), and impaired motor function [226]. The facilitation of optimal motor learning can be achieved by focusing on several factors [227]. The provision of an immersive VR experience offers an enhanced level of motion immersion and illusion, thereby enabling the brain to more effectively discern spatial characteristics and enhance motor function [228,229]. Xiao et al. [230] employ sEMG to initiate virtual ankle movements via the virtual reality-feedback (VRF) system. This system enables the forearm sEMG of a portable armband to facilitate flexible and precise wrist movement recognition, thereby constructing a smooth mapping of movement intentions between sEMG and continuously variable angles. This approach ensures accuracy and real-time performance. Following 15 sessions, 40 patients exhibit notable improvements in body ownership and kinesthetic illusion scores. Additionally, the VRF system’s mechanism of enhancing LE motor function is reflected in the ‘remote effect’ between UE and LE. Specifically, the contraction of muscles in the UE modulates the corticospinal and spinal reflex motor circuits in the LE, which can subsequently influence the motor state of the muscles in the LE. Wearable robots have the potential to facilitate real-time feedback for motor re-learning processes and active motor training. Zhang et al. [231] employ a wearable ankle robot to facilitate sensory-motor rehabilitation in bed, utilizing sensors that are sensitive to initial recovery movement signals and can provide essential real-time feedback to expedite the motor re-learning process. The experimental group exhibit superior outcomes in comparison to the control group, as evidenced by significant improvements in the Fugl-Meyer Motor Assessment of Lower Extremity (FMA-LE) (*p* = 0.007), plantar flexor strength (*p* = 0.009), and active range of motion (*p* = 0.011). Additionally, the experimental group demonstrates an earlier recovery of plantarflexion and dorsiflexion than the control group (*p* < 0.05)

#### 4.2.3. Measures to Improve Sensory Function

The term “sensory integration” is used to describe the brain’s capacity to gather, process, and utilize sensory information. Research findings indicate that approximately 50% of individuals who have experienced a stroke exhibit sensory deficits, particularly in the domains of tactile and proprioceptive discrimination [232]. Many efficacious interventions have been demonstrated to enhance sensory function. These include repetitive sensory discrimination activities, electrical stimulation interventions, thermal stimulation interventions, bilateral simultaneous movements, compression techniques (such as weights and pressure splints), intermittent pneumatic compression, mobilization, and magnetic stimulation. Mirror therapy represents an efficacious therapeutic intervention that tests the five senses [233]. Hsieh et al. [234] employ action observation therapy and mirror therapy to provide visual and proprioceptive feedback of the intact arm, to promote neural reorganization and motor relearning in patients on different afferent inputs and visual feedback. The findings indicate that action observation therapy represents a promising alternative to active control interventions for bilateral arm training in subacute stroke patients. Furthermore, ultrasound has been demonstrated to facilitate the integration of the auditory and sensorimotor systems [235,236], thereby reinforcing and bolstering the impaired proprioceptive system. Raglio et al. [237] devise a music-based ultrasound technique to bolster the patient’s proprioceptive system, allowing the patient to concentrate predominantly on motor outcomes. This approach leads to a notable increase in the FAM-UE (*p* = 0.024). Maximizing the use of the impaired side of the brain facilitates improved functional outcomes. The use of rTMS is more efficacious when initiated within 2–3 months following a stroke, particularly concerning UE recovery [238]. Vink et al. [239] employ a novel inhibitory rTMS paradigm, cTBS, to inhibit healthy side movements and enhance sensory function on the affected side, markedly reducing treatment duration (40 s per session). Consequently, the utilization of cTBS may enhance patient comfort and augment the cost-effectiveness of the intervention.

#### 4.2.4. Methods of Motion Control and Balance

The utilization of RAT has been demonstrated to result in notable enhancements in kinematic parameters and motor function of the limbs [240]. The utilization of robotic devices to generate muscle vibration has been associated with the enhancement of proprioceptive input. This is evidenced by the observation that muscle vibration can facilitate the rapid and significant enhancement of kinesthesia, as well as the facilitation of stroke recovery through cortical plasticity in healthy adults. In a two-phase treatment of 83 patients, Cordo et al. [241] employ the RA motion device (AMES Technology, Inc., Portland, OR, USA) for the distal UE, given that this region is typically more severely impaired than the proximal UE. The implementation of a patient-assisted movement strategy with the device demonstrates enhanced proactivity, resulting in a 10.8-point increase in FM scores in the experimental group, in comparison to a 6.4-point increase in the control group. The advancement of sensors and control algorithms has enabled the development of adaptive controllers for robotic actuators, which are capable of detecting the patient’s locomotor ability in real-time and actively involving the patient in the gait training process. Shin et al. [242] employ an active-mode robot to facilitate gait training and devise a novel neuroimaging experimental paradigm to discern cortical involvement associated with gait events. Ranzani et al. [243] leverage the haptic rendering capabilities of the robot to bolster somatosensory training and assessment, concentrating on functional movement training at the hand level, and observed enhanced clinical performance in patients.

Table 3 lists the design rationale, clinical scales, engineering innovations, target sites, rehabilitation methods, and clinical assessments and outcomes of various subacute phase rehabilitation programs. The subacute period after stroke has attracted a wide range of intensive, task-oriented interventions targeting motor learning, sensory integration and postural control. High-dose conventional therapy, robotic-assisted upper-limb and gait training, modified constraint-induced movement therapy, motor imagery and mental practice, and VR– or game-based programs have all demonstrated short-term improvements in motor function and activities of daily living in selected patient groups. Repetitive, task-specific practice delivered in the first months after stroke can enhance upper-limb recovery, although effect sizes vary and optimal combinations of techniques remain uncertain. Hatem et al. [244] concluded that high-intensity, task-specific training approaches are among the most promising strategies in the subacute phase. However, many trials enroll relatively small samples, often in the range of a few dozen participants, use diverse inclusion criteria and outcome measures, and provide only short-term follow-up, which makes it difficult to compare interventions directly or to determine the durability of gains. As shown in Table 3, the more established approaches—such as high-dose conventional therapy and robotic-assisted training—are supported by randomized trials with samples ranging from several dozen to over 200 participants, whereas newer paradigms including ultrasound-based neuromodulation, BCI-driven training or highly specialized task protocols are still represented mainly by smaller single-center studies (often *n* < 30). Systematic reviews of physical rehabilitation dose and therapy content emphasize that while additional therapy time can improve activity limitations, the amount of extra rehabilitation delivered in routine practice is often far below the levels tested in efficacy trials. From a translational perspective, future research in the subacute phase should focus on designing scalable programs that can deliver high-intensity, individualized training within real-world resource constraints, and on conducting larger, methodologically rigorous trials that compare technology-assisted interventions against best-practice conventional therapy, with sufficient follow-up to assess long-term functional independence and quality of life.

### 4.3. Chronic Phase

Three months after a stroke, the window of neuroplasticity is substantially reduced, thereby rendering spontaneous recovery challenging. Indeed, recovery in the chronic phase, as measured by the Neurological Injury Scale, appears to be approximately 10% of that in the subacute phase. Consequently, at this juncture, it is imperative to introduce intensive neuro- rehabilitation treatments. As patients are discharged from the hospital, there is also a new need for game-based, portable home rehabilitation systems. At this stage, the state of rehabilitation remains the primary consideration and an appropriate rehabilitation system needs to be designed according to available resources and training needs. Secondly, in terms of functional recovery, innovative treatments aimed at improving spasticity, reducing weight-bearing, and improving body control and balance can provide long-term improvement for a wide range of motor deficits and injuries. Finally, addressing cognitive and affective deficits facilitates a rapid return to ADLs.

#### 4.3.1. Demand for Rehabilitation Conditions in Chronic Phase

For patients who live in the community without easy access to healthcare centers or who need to be kept at a physical distance, there is a need for more affordable ways to provide intensive, task-specific treatment in the chronic phase of stroke. Home rehabilitation can fill the gap of insufficient training volume for patients in the chronic phase of stroke [248,249]. For example, TR allows patients to train at home and therapists to help remotely through online communication. Rozevink et al. [250] combine the ArmAssist, a portable robotic device, with a telecare platform to more safely and effectively measure a patient’s active or passive movements, and the Wolf Motor Function Test (WMFT) improves significantly by 3.8 points (*p* = 0.006). It has also been shown that TR using non-robotic serious games equipment at home appears to be more effective for highly motivated, moderately affected chronic stroke patients [251]. Chen et al. [252] integrate an interactive TR exercise game system to improve motor, cognitive, and functional outcomes in stroke survivors, particularly when delivered as a goal-directed program. Furthermore, tDCS is a safe and straightforward method for home-based rehabilitation in patients with access to a paralyzed arm [253].

An emerging trend is to integrate individual active rehabilitation devices within broader digital health infrastructures that support home-based recovery after stroke. Home-based digital technologies for post-stroke rehabilitation are typically multimodal and system-based, combining telerehabilitation platforms, gamified upper-limb training, virtual reality and remote supervision to deliver intensive therapy at a distance [254,255]. For example, Alwadai et al. [256] summarized the impact of various telerehabilitation interventions on motor function, balance, gait, activities of daily living and quality of life, concluding that carefully designed remote programs can improve functional outcomes in stroke survivors. Park et al. [257] systematically reviewed interactive telerehabilitation using VR, game-based systems, smartphone apps and web-based videoconferencing for older adults and neurological populations, and found positive effects on balance and gait when remote monitoring and guidance were provided. Periša et al. [258] proposed a conceptual mathematical model for using AI- and machine-learning–based predictive information to support people with disabilities in smart homes, illustrating how personalized alerts and context-aware assistance can be generated from sensor data. As stroke survivors age and accumulate comorbidities, especially those with disability living in the community, embedding active rehabilitation into these integrated, home-based digital environments may be crucial for sustaining long-term engagement, secondary prevention and functional independence across the continuum of care.

#### 4.3.2. Long-Term Recovery of Motor Function

Spasticity represents a significant obstacle to motor recovery in the rehabilitation of chronic stroke patients. Therapies that passively trigger a reduction in spasticity can, in some cases, reveal the underlying motor potential [259]. In a study conducted by Chang et al. [260], transcutaneous auricular vagus nerve stimulation (taVNS) is administered during robotics training, particularly during the pre-movement planning phase of arm extension exercises. Upon discharge, the subjects exhibit significantly reduced spasticity and an alleviation of spasticity in the wrist and hand. Body weight unloading (BWU) has been proposed as a potential training method for individuals with neurological disorders who experience significant limitations in their ability to walk [261]. A reduction in the patient’s exercise load allows for a greater focus on the quality of the exercise. Skovgaard Jensen et al. [262] devise a dynamic RA weight offloading technique to provide superior training stimulation and sustain functional improvement for individuals with residual impairment in the chronic phase following an ischemic injury. Furthermore, the use of ankle-and-foot orthoses, joints, and RAT has been demonstrated to result in notable enhancements in kinematics and motor function, as evidenced by research findings. Robotic therapy has been demonstrated to enhance trunk stability while concurrently reducing the reliance on compensatory strategies. De Luca et al. [263] conduct a comprehensive series of RA exercises targeting diverse body regions (ranging from the LE to the trunk) to improve core stability through the strengthening of the abdominal, pelvic, and psoas muscles. The experimental group exhibit superior retention of the observed improvement at the three-month follow-up visit. Myoelectrically driven flexion-extension rehabilitation with the aid of a soft manipulator has also been demonstrated to be an effective method for promoting functional recovery of the UE in subjects who have suffered a stroke and who exhibit mild or no spasticity [264].

Regarding limb control and balance, MT facilitates autonomic control of the impaired LE (particularly the ankle) by amplifying the information (visual, motor, and proprioceptive pathways) that determines recovery from more complex neurological dysfunctions. This approach, when combined with VR, can be utilized to enhance motor function in the LE, thereby facilitating recovery of balance, stability, and coordinated gait by training the muscles responsible for these activities. Furthermore, BCI is frequently employed in conjunction with other technologies, including FES and VR. This integration enables users to induce neuroplasticity, thereby enhancing motor function through the real-time detection of MI while they engage in therapeutic activities. Miclaus et al. [265] develop a personalized motor learning paradigm based on VR using MT, whereby the intensity of feedback and training could be systematically manipulated and enhanced. Sebastián-Romagosa et al. [266] demonstrate that UE motor function could be significantly improved by combining MI therapy with VR avatars and FES, thereby providing real-time feedback based on EEG signals from each patient. It is therefore relevant to consider the potential benefits of a multi-faceted approach to the treatment of individuals who have suffered a chronic stroke. This may assist in identifying the optimal solution, to reduce the disability and the burden that is borne by the individual and their family, society more widely, and the health system.

#### 4.3.3. Enhanced Cognitive Functioning

Cognitive and Affective Disorders have a direct impact on the quality of life and independence of affected individuals. There have been identified associations between cognitive impairment, depression, anxiety, and greater dependence on ADLs. Additionally, functional recovery may also be indirectly affected [267,268]. Haire et al. [269] have achieved enhanced psychological flexibility in participants who have suffered a chronic stroke by employing Therapeutic Instru mental Music Performance (TIMP). This may be attributed to multisensory integration and representational consolidation, which are achieved through active practice followed by MI rehearsal. Furthermore, adjunctive stimulation, such as VNS, has been identified as a potential strategy for enhancing post-stroke brain reorganization and promoting the regulation of neurons in the motor cortex, thereby alleviating cognitive deficits.

Table 4 lists the design rationale, clinical scales, engineering innovations, target sites, rehabilitation methods, and clinical assessments and outcomes of various chronic phase rehabilitation programs. Clinically meaningful motor and cognitive gains remain possible months to years after stroke, particularly when rehabilitation is intensive, task-specific and delivered with high repetition or meaningful engagement in daily activities. Long-lasting windows of enhanced neuroplasticity and experience-dependent reorganization have been demonstrated beyond the subacute period, supporting the rationale for continued active rehabilitation in chronic survivors [270]. Robotic training, VR-based therapies, mental practice and combined cognitive–motor programs typically improve specific domains such as upper limb function, balance or mood, but not uniformly outperforming well-delivered conventional therapy or translating into large gains in independence or quality of life across all patients. For example, Soleimani et al. [271] found that adjunctive VR improves upper limb outcomes but with limited superiority over standard care. Many trials remain small, single-center studies with short follow-up and outcome measures focused primarily on impairment or capacity, whereas long-term community participation, self-management and health-related quality of life are less frequently reported, despite evidence that chronic stroke survivors often continue to experience substantial restrictions in these domains [272]. Consistent with Table 4, most technology-assisted rehabilitation programs in the chronic phase enroll between about 10 and 40 participants and follow patients for only a few weeks to months, so current data should be interpreted as early-phase evidence of feasibility and short-term benefit rather than definitive proof of long-term clinical effectiveness. Moreover, substantial heterogeneity in program content, intensity and adherence makes it difficult to isolate the active ingredients of complex interventions. For engineering and clinical design, chronic-phase active rehabilitation should prioritize sustained engagement, integration with home and community environments, and support for self-management and secondary prevention, building on the digital and smart-home infrastructures, rather than relying solely on short, intensive bursts of technology-assisted therapy. Larger pragmatic trials and real-world implementation studies are needed to determine how best to combine, sequence and personalize these technologies to maximize long-term functional gains in chronic stroke survivors.

A meta-analysis of the literature reveals that while numerous studies have examined the efficacy of various rehabilitation modalities, few have considered the fact that patients often have disparate rehabilitation needs at different stages of their recovery. Furthermore, practitioners must consider individualized factors when designing rehabilitation programs. Therefore, in this chapter, we summarize the effects of different rehabilitation methods in various phases of rehabilitation and reorganize the active rehabilitation measures and studies in different phases of post-stroke care. We consider the factors that should be considered when designing a rehabilitation program, such as the rehabilitation conditions, the focus on functional recovery, the evaluation of clinical scales, and so on. We aim to provide a reference for healthcare workers in designing an appropriate rehabilitation program based on the actual situation of their patients.

## 5. Discussion

In the process of designing personalized rehabilitation plans, the individual differences among different patients present a challenge in visualizing patients’ movement intentions, particularly in EEG signals. These signals may be influenced by various internal states, such as attention, fatigue, and motivation, which can affect the reliability of the EEG data. Although BCI is a prevalent tool in numerous active rehabilitation programs and has exhibited substantial rehabilitation outcomes, it may prove challenging for some severely paralyzed stroke patients to attain the requisite performance to utilize BCI. In this regard, Faller et al. [278] put forth a proposal based on an assistive-adaptive system that could assist patients when their BCI performance is inadequate. Secondly, from an engineering perspective, identifying the optimal combination and parameters for multimodal feedback in motor rehabilitation for patients with specific pathologies, such as cognitive impairment, visuospatial neglect, aphasia, dysphagia, spasticity, and motor dysfunction, represents a promising avenue for further research. For instance, the application of multichannel transcranial electrical and magnetic stimulation has been demonstrated to enhance cognitive and emotional functioning in individuals who have experienced a stroke. Similarly, the combination of mechanical stimulation with NMES is an effective approach for addressing motor dysfunction.

Building on the challenges in visualizing movement intentions and accounting for fluctuating internal states, an emerging systems-level approach in neurorehabilitation is the development of AI-based digital patient twins. In the context of stroke, a digital patient twin can be understood as a personalized, dynamically updated and predictive computational representation of a survivor’s neuro-musculoskeletal and functional status [279]. Chen et al. [280] proposed a digital-twin-based patient evaluation framework for stroke rehabilitation, in which a motor control model is continuously updated using kinematic performance data so that the twin can be used to plan customized training tasks and reduce uncertainty in therapy design. Lauer-Schmaltz et al. [281] built a human digital twin system for upper-limb stroke rehabilitation that uses the ETHICA methodology to monitor patient status and adapt exoskeleton assistance and game difficulty in real time. In parallel, recent reviews have highlighted how patient-specific digital twins in healthcare can leverage artificial intelligence to fuse clinical scales, imaging, biosignals and electronic health-record data into dynamic models that support monitoring, risk prediction and individualized treatment planning [282,283,284,285]. Generative AI is increasingly seen as a key enabler in this context: Vidovszky et al. [286] reviewed AI-generated digital twins and discussed how large generative models can help construct and update patient-specific twins from heterogeneous healthcare data, while Vengathattil et al. [287] outlined how GenAI-driven digital twins may turn static representations into adaptive, predictive and prescriptive systems for personalized care.

Therefore, AI-based digital patient twins can continuously assimilate biosensor-derived information and clinical outcomes to update a virtual representation of each patient, and then use this representation to suggest adjustments in the choice, timing and intensity of active rehabilitation interventions—whether robot-assisted training, BCI-mediated feedback, or home-based telerehabilitation—across the acute, subacute and chronic phases of recovery. In turn, the effectiveness of such digital-twin architectures critically depends on robust real-time intent detection and reliable measurement of internal states such as attention and fatigue.

### 5.1. Challenges of Real-Time Intent Detection

A key advancement in active rehabilitation over traditional stroke rehabilitation is the incorporation of patient intention detection into the training session, which significantly enhances the real-time accessibility of sensory feedback on the patient’s immediate motor intentions. In the context of stroke rehabilitation, the real-time pairing of motor intentions with associated feedback is of critical importance for the induction of neuroplasticity. Two questions therefore warrant further investigation: (i) are there varying requirements for the maximum delay between intention and feedback across different application scenarios, and (ii) does reducing this delay consistently lead to clinically meaningful improvements in rehabilitation outcomes? Existing work indicates that when the detection and feedback devices are operating optimally, the response latency of the system cannot exceed a certain threshold without compromising the efficacy of rehabilitation. For instance, enhancing the real-time capability of intent detection allows for a greater temporal allowance for multi-degree-of-freedom feedback.

Two families of architectures currently appear particularly promising for meeting these latency constraints in practice. For patients with mild-to-moderate impairment who retain some voluntary movement, hybrid pipelines that combine surface EMG with peripheral status signals and implement lightweight feature extraction and classification on embedded processors can provide millisecond-level timing while still capturing rich information about effort and context. In contrast, for patients with severe paresis or minimal residual movement, central neural signals such as EEG or fNIRS must carry most of the intention information; here, pairing event-related desynchronization or hemodynamic responses with FES, robotic assistance or VR-based feedback, and exploiting predictive, patient-specific decoders, may enable clinically acceptable delays even though the raw signals are slower and noisier.

Across both groups, wiring detection and actuation into tightly integrated wearable modules, minimizing data transmission overhead and using event-triggered rather than continuous control logic are key engineering strategies for closing the intent–feedback loop within the temporal windows suggested by experimental studies of activity-dependent plasticity and behavioral learning. In this context, electromagnetic approaches that unify detection and feedback functions may serve as one concrete direction for future minimal-delay active rehabilitation systems.

### 5.2. Fatiguing Problems Caused by Feedback Interventions

During the rehabilitation process, the provision of continuous feedback from assessments may result in patient fatigue. For instance, the use of continuous EMG control may serve to reinforce pathological movements rather than facilitate the recovery of normal movement patterns. Consequently, further research is required to ascertain how pathological movements associated with EMG-controlled rehabilitation can be effectively avoided. An important direction is the development of fatigue-aware control policies that monitor changes in EMG and kinematic patterns over time and dynamically adjust stimulation dosage, robotic assistance and task complexity. Such adaptive scheduling, combined with home-based monitoring of adherence and motivation, could help ensure that intensive active rehabilitation remains tolerable and sustainable over weeks to months, rather than only during short experimental protocols.

### 5.3. Considerations for Finding the Best Rehabilitation Program

In the search for the optimal rehabilitation program for stroke, it is possible to consider the active rehabilitation methods that have been demonstrated to be effective for other diseases. For instance, in the treatment of Parkinson’s disease, a neurological disorder characterized by tremor, motor dysfunction, gait disturbances, and cognitive deficits, nonpharmacological interventions, such as DBS, have demonstrated the potential to assist patients suffering from drug-resistant tremor, symptomatic exacerbation during discontinuation of medication, and motor dysfunction, which can be clinically significant for stroke hemiplegic patients with motor recovery. In a review of various VR treatments in psychiatry, Park et al. [288] find that VR exposure therapy is particularly effective for anxiety disorders, eliciting realistic responses to fearful stimuli. Similarly, simulated imagery exposure therapy has been shown to have significant benefits for patients with phobias and post-traumatic stress disorder (PTSD). Therefore, VR exposure therapy has the potential to complement cognitive and social skills training for stroke patients, thereby improving their quality of life. Furthermore, given the diverse manifestations of stroke disease, including aphasia, hemiparesis, and cognitive deficits, it is essential to consider the sequence of treatments for different pathologies when designing or screening treatment programs. For instance, it is necessary to determine whether motor training for the treatment of hemiplegia will have a detrimental impact on cognitive recovery and whether cognitive training can be integrated concurrently with language function enhancement [289]. Consequently, the design of trials to validate the efficacy of screened rehabilitation programs represents a crucial aspect of clinical practice.

### 5.4. Designing Protocols Based on Physiological Foundations

To design effective active rehabilitation programs for stroke patients, it is imperative to ground both hardware design and protocol development in the foundational principles of neuroplasticity. Achieving optimal therapeutic outcomes necessitates an integrative approach that deeply incorporates the physiological mechanisms of recovery, such as sensorimotor circuit dynamics, synaptic plasticity, and the temporal patterns of adaptive responses to neural injury. Building on the neurophysiological foundations allows for the development of devices and protocols that not only address patient-specific needs but also actively stimulate neuroplastic engagement. By harmonizing technological innovation with these core biological principles, active rehabilitation programs can make full use of critical recovery windows, foster patient motivation and engagement, and ultimately drive more profound functional improvements.

### 5.5. Translating Clinical Needs into Engineering Requirements

Building on these physiological and technological considerations, it is useful to make the “bridge” between clinical and engineering perspectives as explicit as possible. In this subsection, we therefore use representative scenarios to illustrate how typical clinical goals can be translated into concrete sensing and feedback requirements, and how emerging capabilities create new opportunities for active rehabilitation across stroke phases.

#### 5.5.1. Example 1—Reducing Distal Spasticity and Enabling Task-Specific Hand Use in Chronic Stroke

In chronic stroke survivors, a common clinical goal is to reduce wrist–hand flexor spasticity while restoring active extension to support grasp-and-release in daily activities. NMES/FES combined with task-specific training can reduce spasticity and improve distal motor function in selected patients. From this starting point, several engineering requirements follow. First, the stimulation device should provide multi-channel output and flexible control of pulse width, frequency and intensity within safety limits, so that target muscle groups can be activated while avoiding excessive discomfort or clonus. Second, a trigger signal is needed that reliably reflects the patient’s intention to open the hand or extend the wrist; for mildly to moderately impaired patients this can be derived from residual surface EMG or peripheral status signals, and the detection pipeline must operate with low latency (on the order of a few hundred milliseconds) to preserve temporal pairing between intention and stimulation. Third, the control logic should incorporate safeguards against pathological co-contraction and fatigue, for example, by limiting duty cycles, adapting intensity based on recent output, and exposing parameters that can be tuned according to spasticity severity and functional goals. These considerations translate the abstract aim of “reducing spasticity and enabling functional hand use” into a concrete set of specifications for channel configuration, timing, triggering and user interfaces.

#### 5.5.2. Example 2—Providing Intention-Driven Feedback in Patients with Minimal Residual Movement

For patients with severe hemiparesis who retain consciousness but have little or no visible voluntary movement, the clinical challenge is to move beyond purely passive mobilization and provide genuinely active, intention-driven rehabilitation. EEG- or fNIRS-based BCIs coupled with FES or robotic-assisted training can enable intention-driven feedback in such patients, leading to short-term functional gains and measurable changes in cortical activation. Translating this into engineering requirements, the sensing module must extract intent-related features (for example, event-related desynchronization patterns over sensorimotor areas) in real time, with classifiers that can be adapted across sessions to mitigate “BCI-inefficiency” in a substantial subset of users. The actuation module—whether FES, an exoskeleton or a robotic end-effector—needs to deliver joint trajectories or muscle activation patterns that are time-locked to detected intentions within a narrow temporal window, while respecting safety constraints for frail patients. Closed-loop control should incorporate feedback on both performance (e.g., achieved range of motion, trajectory quality) and neurophysiological state (e.g., attentional markers), enabling the system to adjust task difficulty and assistance levels over time. In this way, the general goal of “making severely impaired patients active participants in their own therapy” becomes a concrete specification for multi-level sensing, decoding and actuation.

#### 5.5.3. Example 3—Delivering High-Dose, Sustainable Practice in Home and Community Settings

In the chronic phase, many community-dwelling stroke survivors require long-term, affordable rehabilitation outside the hospital. Home-based telerehabilitation and VR-enhanced programs can be feasible and beneficial for selected patients, although adherence and long-term engagement remain variable. From an engineering perspective, this scenario leads to a distinct set of requirements. Sensing should rely on robust, low-cost wearable devices—such as inertial measurement units and surface EMG—to monitor both movement intention and movement quality in daily life contexts. User interfaces need to be simple enough for patients and caregivers to operate independently, while allowing clinicians to remotely configure programs and review performance data within their existing workflow. Software architectures must handle intermittent connectivity and ensure data security. Rehabilitation content should be designed to adapt task difficulty, feedback richness and narrative elements to patients’ cognitive and emotional profiles, with built-in mechanisms to monitor fatigue and safety (for example, fall risk alerts). These specifications translate the ambition of “sustained, enjoyable home-based practice” into concrete design targets for hardware, algorithms and clinical integration.

The examples illustrate how phase-specific and severity-specific clinical goals can be mapped onto sensor, decoder, actuator and interface design choices, and how recent advances in FES, BCI and telerehabilitation technologies can be aligned with these requirements to support active rehabilitation across the stroke continuum.

## 6. Conclusions

In this paper, a comprehensive analysis of the physiological basis underlying active rehabilitation is presented. It systematically categorizes the devices and techniques utilized in the two primary aspects of active rehabilitation, namely, intention detection and sensory feedback. Finally, it presents a systematic approach to the design principles and potential therapeutic effects of combining various detection and feedback modalities during different rehabilitation periods following a stroke. Our findings indicate that active rehabilitation techniques have the potential to enhance neuroplasticity mechanisms in the brain. We hope that this study will assist frontline healthcare professionals in promoting the implementation and dissemination of active rehabilitation modalities in clinical practice, thereby improving the rehabilitation outcomes for stroke patients.

## Figures and Tables

**Table 1:** Reviews of the application of active rehabilitation techniques in stroke rehabilitation.

Reviews	Content	Contribution
[8]	Reviewing the state-of-the-art in the field of stroke rehabilitation with a multidisciplinary approach.	Analyzing the integration of exergames, TR, and robotic systems in enhancing motor recovery.
[9]	Providing a systematic analysis of VR, RAT, and TR’s impact on post-stroke UE rehabilitation.	(1) Evaluating the relative effectiveness and optimal timing of new technologies versus traditional therapies at different stages of stroke rehabilitation. (2) Assessing the influence of intervention design, rehabilitation duration, and severity of motor impairments on the outcomes of rehabilitation technologies.
[10]	Analyzing VR advancements in stroke rehabilitation, focusing on therapeutic potential and clinical integration for UE recovery.	(1) Identifying and discussing the integral features of VR systems, such as multisensory feedback and adaptive difficulty levels. (2) Proposing a patient-centered approach to VR utilization in clinical settings.
[11]	Reviewing robotic advancements in hand rehabilitation post-stroke, evaluating technological integration and therapeutic efficacy.	Based on user needs, the technical status quo and shortcomings of functional hand rehabilitation robots are sorted out to point out the direction for technical development.
[12]	Synthesizing development in EEG and EMG applications for poststroke rehabilitation.	Highlighting the technological advancements in using EEG and EMG for cognitive intention recognition, motor imagery (MI), and function rehabilitation devices, paving the way for more effective and personalized stroke rehabilitation strategies.
[14]	Presenting recent progress in EEG-based BCIs for post-stroke rehabilitation, emphasizing neural plasticity leveraging and motor function restoration.	(1) Evaluating the integration of EEG with MI and VR for enhanced stroke rehabilitation outcomes. (2) Discussing the convergence of machine learning techniques with EEG data for improved decoding of user intentions in BCI systems.
[15]	Reviewing promotions in holistic BCI system for motor, cognitive, and affect rehabilitation.	Interrelationships between joint motor, cognitive, and affective functioning to explore integrated treatment options that target the synergistic effects of these independent interventions.
[16]	Synthesizing progress in MI-based BCI systems for post-stroke rehabilitation.	(1) Evaluating MI-BCI strategies integrating functional electrical stimulation (FES), robotics, and VR for UE recovery. (2) Discussing future smart rehabilitation systems incorporating flexible electronics.
[17]	Systematic evaluation of immersive VR’s role in post-stroke rehabilitation.	Exploration of the role of fully immersive virtual reality (FIVR) in enhancing motor recovery and rehabilitation programs after stroke.
[18]	Summarizing the role of multimodal sensing technologies in enhancing stroke motor rehabilitation through real-time feedback mechanisms.	Examining the integration of various sensors (e.g., EMG, EEG) in rehabilitation devices to optimize patient outcomes.
[19]	Providing a systematic review of wearable sensors and machine learning applications in post-stroke rehabilitation assessment.	Providing a comprehensive review of wearable sensor technologies and machine learning approaches used in post-stroke rehabilitation, from feature engineering to classification.
[20]	Exploring augmented reality (AR) for post-stroke rehabilitation.	Investigating the possibility of applying AR in more contextually relevant environments to enhance motor learning and generalize to other tasks.
[21]	Providing a systematic review of technology-based compensation assessment and detection methods for UE activities in stroke survivors.	The integration of multiple detection techniques and algorithms is emphasized to improve the accuracy and effectiveness of compensated detection.
[22]	Reviewing advancements in neurofeedback for post-stroke rehabilitation, emphasizing adapted approaches.	Delineating neurofeedback’s role in modulating brain activity for motor recovery.
[23]	Synthesizing recent progress in robotic biofeedback for post-stroke gait rehabilitation.	Investigating the integration of biofeedback systems with assistive devices in gait rehabilitation.
[13]	Summarizing advancements in real-time BMF systems for sports and rehabilitation.	(1) Evaluating sensor technologies and processing methods for motion analysis. (2) Examining the integration of feedback modalities in wearable systems for performance enhancement and recovery.
[24]	Systematic examination of sensorimotor rhythm-based BCIs for UE rehabilitation post-stroke.	(1) Synthesizing motor task paradigms and feedback modalities in BCI-assisted rehabilitation. (2) Evaluating the clinical relevance and neurophysiological impact of BCI training on motor recovery.
[25]	Reviewing advancements in BCI-robotic systems for post-stroke hand rehabilitation.	Concentrating on the rehabilitation of fine motor skills.
[26]	Investigating the potential for robot-assisted (RA), VR-based rehabilitation and automated assessment.	Quantitative analysis of automated assessment methodologies with clinical validation.
[27]	Demonstrating the feasibility and safety of a bedside BCI system for acute/subacute stroke rehabilitation.	(1) Introducing real-time feedback of sensorimotor rhythms through a portable BCI system. (2) Successfully conducting bedside BCI training trials with hemiplegic stroke patients in the acute/subacute phase, paving the way for larger clinical studies to evaluate its clinical efficacy.

**Table 3:** Rehabilitation program in the subacute phase after stroke.

Studies	Design Basis	Main Clinical Scales	Engineering Innovation	Target Parts	Rehabilitation Methods	Number of Patients	Duration of Intervention	Main Results
[222]	Economic sustainability	FMA *	The efficacy of robotic UL therapy is evaluated using a group of 4 devices	UE *	Standardized UE robotic rehabilitation	111	30 T *, 22 H *	Significantly improved UE * motor function, activity and participation.
[223]	Treatment time	FMA *	N/A *	UE *	A gravity-assisted, games-based therapy system	215	4 W *, 20 T *, 20 H *	The mean improvement in FMA-UE * score is 13.32 (±9.03) in the experimental group and 11.78 (±8.84) in the control group.
[224]	Intensity of tasks	FM-UE *	Providing high-dose, high-intensity, motion-quality focused therapy	UE *	NAT	24	30 T *, 30 H *	No significant improvement in FMA-UE * scores, but significant benefit on ARAT.
[225]	Intensity of tasks	FMA-UE *, total pROM *, MAS *-Shoulder, MAS *-Elbow	N/A *	UE *	Planar end-effector robots	53	6 W *, 30 T *	Significant improvement in FMA-UE * (*p* < 0.001).
[230]	High-level immersion and motor learning strategies	SSQ *, VAS *, 7-point LS *	A novel data-driven methodology for precise intent mapping	Limbs	sEMG + VR	40	15 T *	Patients who complete the VR condition have significantly higher body ownership and kinesthetic illusion scores.
[231]	Real-time feedback to facilitate the motor re-learning process	FMA-LE *, MAS *	Real-time feedback-guided motor re-learning training, combining passive and active motor training	Ankle	Wearable Ankle Robot	18	5 T/W *, 50 M/T *	Improvements in FMA-LE * (*p* = 0.007), plantar flexor strength (*p* = 0.009), and active range of motion (*p* = 0.011) are greater.
[234]	Proprioceptive feedback	FM *	N/A *	UE *	Action Observation Therapy and Mirror Therapy	21	N/A *	Improvements in functional independence measures are greater in the movement observation therapy than in the other 2 groups. The mirror therapy group showed the least improvement.
[237]	Proprioceptive feedback	FMA-UE *, Box and Block Test, MAS *	Musical elements are automatically associated with the patient’s movements and focus their attention	UE *	A music-based approach to ultrasound	65	N/A *	Improvement in total FMA-UE * (*p* = 0.024).
[239]	Increased use of the affected side	FM *	The relatively brief duration of cTBS treatments enhances patient comfort and improves the cost-effectiveness of the intervention.	UE *	Inhibitory rTMS	60	10 T/D *	The mean difference in arm test scores relative to baseline is 9.6 points and the length of hospital stay is 18 days shorter than in the control group.
[241]	Motion control	FMA-UE *	Muscle vibration generation, patient-assisted equipment movement	UE *	The robotic-assisted motion device	83	2 phases	FM scores increase by 10.8 in the experimental group and 6.4 in the control group.
[242]	Balance of the trunk	FAC *, MAS *, FMA *	A new experimental paradigm in neuroimaging	LE * and gait	Active mode RA gait training	14	8 W *, 16–24 T *, 8–12 H *	FAC *, MAS *, and FMA-LE * significantly improve (*p* < 0.05).
[243]	Motion control	FMA-UE *	Tactile rendering capabilities of robots	Hands	RAT	33	4 W *, 15 T *, 11 H *	The RA/conventional treatment group improves on the FMA-UE * by 7.14/6.85, 7.79/7.31 and 8.64/8.08 points, respectively.
[245]	Provision of intensive and specific training	MI-AL *, mBI *, WHS *	Inducing coordinated multisensory motor control stimuli and providing subjects with proprioceptive input during limb loading	LE *	Overground Robot-Assisted Gait Training (o-RAGT)	75	5 T/W *, 1 H/T *	The scores of each scale increase.
[246]	Enhancement of motor learning fun	Resting-state fMRI, FMA-UE *	Progressive task complexity and visual and auditory feedback about successful movement	UE *	Mirroring neuron VR Rehab (MNVR-Rehab)	8	2 W *, 8 T *, 8 H *	Patients show a significant improvement in their FMA-UE * scores (*p* < 0.042).
[247]	Strength and muscle coordination	BBS *, FIM *	N/A *	Muscle Synergy	FES-augmented cycling	9	3 W *, 15 T *, 21 H *	Good associations between biomechanical indices and clinical outcomes (Spearman’s coefficient > 0.65) and gait speed (Spearman’s coefficient ≥ 0.9).

* mBI—The modified Barthel Index, pROM—passive Range of Motion, FAC—Functional Ambulation Category, FIM—Functional Independence Measure, LS—Likert Scale, MAS—Modified Ashworth Scale, MI-AL—The Motricity Index of the Affected lower Limb, SSQ—The Simulator Sickness Questionnaire, VAS—Visual Analog Scale, WHS—The Walking Handicap Scale, N/A—not available, M—Minute(s), D—Day, T—Time, W—Week, H—Hour(s).

**Table 4:** Rehabilitation program in the chronic phase after stroke.

Studies	Design Basis	Main Clinical Scales	Engineering Innovation	Target Parts	Rehabilitation Methods	Number of Patients	Duration of Intervention	Main Results
[250]	Rehabilitation conditions	FMA-UE *	Safer and more effective portable systems	UE *	Robotic devices ArmAssist + telecare platform	10	6 W, 18 H *	WMFT * significantly improves by 3.8 points (*p* = 0.006).
[252]	Rehabilitation conditions	BBS *	First system integration of related hardware	UE *	Interactive TR exergaming system	30	4 W, 12 T *	BBS scores improve significantly in both groups (control group: *p* = 0.01, effect size = 0.49; experimental group: *p* = 0.01, effect size = 0.70).
[253]	Rehabilitation conditions	FMA, Wolf Motion Function Test, Timed Up and Go Test	N/A	Limbs	Dual-hemispheric tDCS (dual-tDCS)	24	4 W, 12 T, 12 H *	Improvement in FMA score in active group, lasting at least 1 month.
[260]	Improvement of spasticity	FMA-UE *, Modified Tardieu Scale	Current selectivity in closed loops during visual cues for actively assisted stretching movements	UE *	taVNS + RAT	36	3 W, 9 T, 9 H *	The active taVNS * group has significantly lower spasticity of the wrist and hand at hospital discharge.
[262]	Weight reduction	FMA-LE *	Dynamic unloading	LE *	Robot-based BWU technology	34	24 W, 2 T/W *	Contribution to the scientific literature on BWU efficacy.
[263]	Enhanced stability	BBS, Trunk Impairment Scale, MMSE *	Different areas of the body and various postures can be trained	Trunk balance	RAT	30	5 W, 15 T, 11 H *	The experimental group shows better-improved retention at 3 months follow-up.
[264]	Motion Recovery	ARAT, FMA-UE, MAS, MAGS *	High compliance and low stiffness with flexion and extension assist torque	Hands	Myoelectrically driven soft manipulator training	16	20 T, 20 H *	ARAT *(increase of 2.44, *p* = 0.032), FMA-UE (increase of 3.31, *p* = 0.003), and maximal voluntary grip strength (increase of 2.14 kg, *p* < 0.001).
[265]	Motion Recovery	FMA-LE *	Systematic manipulation and improvement of the intensity of feedback and training	LE *	VR + MT	59	10 T, 12 H *	There is a significant difference in the clinical status of range of motion and muscle strength between the experimental and control groups before and after treatment (*p* < 0.001).
[266]	Motion Recovery	FMA-UE *	Multimodal integration	UE *	MI + VR + FES	51	25 T *	UE motor function significantly improves by 4.68 points (SD = 4.92).
[269]	Enhanced Cognition	TMT-Part B, DST, MAACL-R, GSE *	Neurological Music Therapy (NMT) Techniques	UE *	TIMP	30	3 W, 9 T, 6 H *	Enhanced psychological flexibility.
[273]	Motion Recovery	N/A *	N/A *	UE *	Epidural stimulation of the cervical spinal cord	2	N/A *	Grip strength + 40%, speed + 30% to + 40%.
[274]	Motion Recovery	FMA-UE, ARAT, MOCA, SIS-16, pROM *	Enhanced extensor activation based on existing systems	UE *	An EMG-based variant of our REINVENT VR neurofeedback rehabilitation system	4	7 T, 7 H *	SIS-16 significantly improves (*p* = 0.011), participants significantly improve their ability to maintain constant levels of activation in the wrist flexors and extensors and demonstrate enhanced 12–30 Hz cortical muscle coherence.
[275]	Trunk balance	The Balancing System SD, the GAITRite System	Reduces the burden on weight-bearing joints and the risk of falls, and provides resistance in multiple directions of motion	Gait	Underwater Treadmill Gait Training	22	4 W, 20 T, 10 H *	Static balance scores improve from 1.16 ± 0.32 to 0.49 ± 0.17 ratings (*p* < 0.00) and dynamic balance scores improve from 3.57 ± 1.45 to 1.78 ± 0.88 ratings (*p* < 0.00).
[276]	Enhanced Cognition	MMAS *, Brunnstrom Stages of Recovery test	The game is designed using the Unity environment, supports Kinect, and does not require the use of any wearable devices.	UE *	VR	10	4 W, 12 T *	Games have positive effects on the horizontal abduction of the shoulder (16.26 ± 23.94, *p* = 0.02), horizontal adduction of the shoulder (59.24 ± 74.76, *p* = 0.00), supination of the wrist (10.68 ± 53.52, *p* = 0.02), elbow flexion (0.1 ± 1.5, *p* = 0.00), and wrist flexion (0.06 ± 1.34, *p* = 0.03).
[277]	Enhanced Cognition	FMA-UE *, WMFT *	N/A *	UE *	VNS combined with task-specific rehabilitation	108	6 W, 18 T *	47% of patients in the VNS group had a clinically meaningful response to the FMA-UE * score, with improvements in multiple measures of arm function 2–3 times higher than in the control group.

* DST—The Digit Span Test, GSE—The General Self-Efficacy Scale, MAACL-R—The Multiple Affect Adjective Checklist-Revised, MAGS—Maximum Autonomous Grip Strength, MMAS—Modified Motor Assessment Scale, MMSE—Mini-mental State Examination, MOCA—Montreal Cognitive Assessment, SIS-16—16-question Stroke Impact Scale, TMT—The Trail Making Test, W—Week, T—Time, H—Hour(s), N/A—Not available.

## Data Availability

No new data were created or analyzed in this study. Data sharing is not applicable to this article.
